# Climatic and Hydrological Changes Drive Contemporary Fire Regime Transitions in the World's Largest Wetland

**DOI:** 10.1111/gcb.71089

**Published:** 2026-09-04

**Authors:** Lucas Barros‐Rosa, Pierre Girard, Priscila Lemes de Azevedo Silva, Fabio de Oliveira Roque, Mark Johnson, Christine Strüssmann, Thadeu Sobral de Souza, Renata Libonati, Liana O. Anderson, Cátia Nunes da Cunha, Geraldo Alves Damasceno‐Júnior, Walfrido Moraes Tomas, Lúcia Mateus, Fernando A. da Costa Fernandes, Daniela de Oliveira Maionchi, Junior Gonçalves da Silva, Higo J. Dalmagro, Jerry Penha

**Affiliations:** ^1^ Institute for Resources, Environment and Sustainability The University of British Columbia Vancouver British Columbia Canada; ^2^ National Institute for Science and Technology in Wetscapes Manaus Amazonas Brazil; ^3^ Department of Botany and Ecology, Institute of Biosciences Federal University of Mato Grosso Cuiabá Mato Grosso Brazil; ^4^ Institute of Biosciences Federal University of Mato Grosso Do Sul Campo Grande Mato Grosso do Sul Brazil; ^5^ Faculty of Veterinary Medicine Federal University of Mato Grosso Cuiabá Mato Grosso Brazil; ^6^ Department of Meteorology Federal University of Rio de Janeiro Rio de Janeiro Rio de Janeiro Brazil; ^7^ Tropical Ecosystems and Environmental Sciences Lab, Earth Observation and Geoinformatics Division National Institute for Space Research (INPE) São José dos Campos São Paulo Brazil; ^8^ Brazilian Agricultural Research Corporation (Embrapa) Corumbá Mato Grosso do Sul Brazil; ^9^ Department of Atmospheric Sciences University of São Paulo São Paulo São Paulo Brazil; ^10^ Graduate Program in Environmental Physics Federal University of Mato Grosso Cuiabá Mato Grosso Brazil; ^11^ Graduate Program in Environment and Regional Development Uniderp Campo Grande Mato Grosso do Sul Brazil

**Keywords:** burned area, drought, fire regime, fire seasonality, flood pulse, hydroclimate, Pantanal

## Abstract

Tropical wetlands have historically been resilient to fire, but ongoing climate and hydrological changes are undermining this stability. Here, we provide robust multi‐decadal evidence (1985–2024) of structural changes in the contemporary fire regime of the Pantanal, the world's largest wetland, associated with hydroclimatic disruption. We investigated changes in burned area, mean fire size, and fire seasonality using hydrological, climatic, and fuel‐condition predictors. To identify the major drivers of fire dynamics, we applied trend analyses and Elastic Net models across multiple temporal and spatial scales. Although no significant monotonic trend was detected for annual burned area across the entire time series, change point analysis identified 2014 as the onset of a significant upward trend. Mean fire size exhibited a significant long‐term upward trend across the full period, which intensified after 2018, suggesting larger fire sizes in recent years. Across models, reduced precipitation, shorter seasonal wetland flood pulses, and higher atmospheric and fuel dryness emerged as the strongest predictors of burned area and fire size. These changes have expanded the duration of the fire season, particularly in the northern and central Pantanal, by delaying the onset of rains and shortening the flood pulse period. Our findings suggest a weakening of the hydroclimatic coupling that has regulated ignition and fuel availability in the Pantanal. Broader environmental changes in the upper basin and regional climate may be reinforcing this transition. These results highlight the Pantanal's increasing vulnerability to drought‐driven fires and underscore the urgent need for integrated water and fire management strategies to preserve the resilience of the Pantanal and other tropical wetlands under intensifying global change pressures.

## Graphical abstract

Long‐term fire data (1985–2024) and integrated modeling reveal a climate and hydrology‐driven transition toward larger fires, increased burned area, and a longer fire season, indicating the emergence of a new fire regime in the Pantanal.

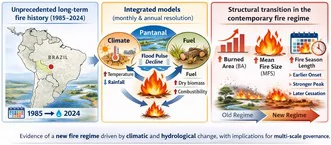

## RESUMO

As áreas úmidas tropicais têm sido historicamente resilientes ao fogo, mas as contínuas mudanças climáticas e hidrológicas vêm comprometendo essa estabilidade. Aqui, fornecemos evidências multidecadais robustas (1985–2024) de mudanças estruturais no regime contemporâneo de fogo do Pantanal, a maior área úmida do mundo, associadas à perturbação hidroclimática. Investigamos alterações na área queimada, no tamanho médio do incêndio e na sazonalidade dos incêndios utilizando preditores hidrológicos, climáticos e de condição do combustível. Aplicamos análises de tendência e modelos Elastic Net em múltiplas escalas temporais e espaciais para identificar os principais fatores determinantes da dinâmica de fogo. Embora nenhuma tendência monotônica significativa tenha sido detectada para a área queimada ao longo de toda a série temporal, a análise de ponto de mudança identificou 2014 como o início de uma tendência significativa de aumento. O tamanho médio do incêndio exibiu uma tendência significativa de aumento ao longo de todo o período, a qual se intensificou após 2018, sugerindo áreas queimadas contíguas maiores em anos recentes. Entre os modelos, a redução da precipitação, a menor duração dos pulsos sazonais de inundação e o aumento do ressecamento atmosférico (com elevação do déficit de pressão de vapor) emergiram como os preditores mais fortes da área queimada e do tamanho do incêndio. Essas mudanças expandiram a duração da estação de fogo, particularmente no Pantanal Norte e Central, ao atrasar o início das chuvas e encurtar o período do pulso de inundação. Nossos achados sugerem um enfraquecimento do acoplamento hidroclimático que historicamente regulou a ignição e a disponibilidade de combustível no Pantanal. Mudanças ambientais mais amplas na parte alta da bacia e no clima regional podem estar reforçando essa transição. Esses resultados destacam a crescente vulnerabilidade do Pantanal a incêndios impulsionados pela seca e sublinham a necessidade urgente de estratégias integradas de gestão de água e fogo para preservar a resiliência do Pantanal e de outras áreas úmidas tropicais sob pressões crescentes de mudanças globais.

## 1 Introduction

Large‐scale wildfires have become a growing threat to ecological and social stability, with devastating effects on people, biodiversity, and ecosystem services (Hurteau et al. 2014; Balch et al. 2015; Fann et al. 2018; Kelly et al. 2020). Tropical wetlands, historically resilient to fire, are now under increasing fire risk due to climate change and land‐use change pressures (Convention on Wetlands 2025). This risk is primarily manifested through alterations in the fire regime, a concept that encompasses factors such as frequency, size, and crucially, the seasonality of fires (Archibald et al. 2018; Pausas and Keeley 2019). Understanding these metrics is a fundamental and pressing concern, as they allow the identification of structural changes that can lead to a major ecosystem reorganization (Faria et al. 2021). Therefore, the analysis of the totality of the fire regime in vulnerable ecosystems is urgently needed to develop effective conservation and adaptive management strategies (McWethy et al. 2019).

The Pantanal, the world's largest continuous wetland, provides a critical system for understanding fire‐regime change because its flammability is governed not only by climate factors, but also by a seasonal flood pulse (Junk and Cunha 2012; Bergier et al. 2018; Damasceno‐Junior et al. 2021). By regulating fuel exposure, limiting fire spread, and maintaining a fine‐scale spatial mosaic of different vegetation formations, this water‐mediated mechanism historically modulated fire behavior (Pott and da Silva 2015; Aoki et al. 2021; Damasceno‐Junior et al. 2021; Pivello et al. 2021). Consequently, contemporary transitions in fire activity signify more than an increase in burned area, they raise critical questions about potential disruptions to the hydroclimatic feedbacks that underpin wetland resilience (Damasceno‐Junior et al. 2025; Pereira et al. 2025).

Recent extreme fire events highlight these growing vulnerabilities. The unprecedented 2020 fires, which burned nearly one‐third of the biome, followed by the 2024 events, affected habitats historically protected by seasonal inundation, triggering massive biodiversity loss (Libonati et al. 2020; Marengo et al. 2021; Garcia et al. 2021; Tomas et al. 2021; Belém et al. 2026). Beyond ecological damage, altered fire dynamics pose risks to the region's socio‐ecological system, including traditional cattle‐ranching, local livelihoods, public health, and the nature‐based economy (Tortato et al. 2017; Tomas et al. 2019; Lorenz et al. 2024; Botelho et al. 2025). Given the biome's vast biomass stocks and high ecological heterogeneity, determining whether these extreme events signal a structural transition in fire regimes is critical for assessing the vulnerability of tropical wetlands under intensifying hydroclimatic stress (Page et al. 2002; Convention on Wetlands 2025; Damasceno‐Junior et al. 2025). Thus, the Pantanal serves not merely as a regional conservation concern, but as a model system for diagnosing how climate and hydrological‐driven degradation reshapes wildfire dynamics in large‐scale floodplains.

Despite the urgency in understanding this crisis, fire science in the Pantanal presents methodological gaps that hinder a clear diagnosis of long‐term regime dynamics. Fire regimes are typically described by multiple components that characterize temporal and spatial patterns of fire occurrence. In this study, we define a fire regime transition as a sustained structural change in key regime components (burned area, fire size, and seasonality) that exceeds expected interannual variability, potentially indicating a reorganization of system dynamics (Scheffer et al. 2001; Scheffer and Carpenter 2003). While fire regimes can be described by numerous parameters, we focus on these core spatio‐temporal metrics as they are most representative of structural changes in fire dynamics (Krebs et al. 2010). Identifying such transitions is often limited by studies that analyze only one component of the fire regime or rely on short time series (e.g., MODIS) available only after the early 2000s (Kumar et al. 2022; Veiga et al. 2025). This restricted temporal scope hinders the detection of structural changes and the influence of slow‐moving modulators such as the flood pulse. Therefore, multi‐decadal records (e.g., Landsat‐based) are essential to identify evidence of contemporary departures from previously observed fire regime variability. Although the 1985–2024 Landsat record represents a relatively short period in a climatic context, it constitutes the longest consistent satellite‐based reconstruction of fire activity currently available for the Pantanal. This interval also encompasses recent catastrophic wildfire events, making it suitable for detecting contemporary changes in fire regime dynamics.

To address this gap, here we use a 40‐year historical series (1985–2024) and integrated modeling of different temporal resolutions (monthly and annual) to investigate the contemporary fire regime transition in the Pantanal and its main drivers. We hypothesize that recent climatic and hydrological changes observed over the last four decades (Caballero et al. 2025; Nunes et al. 2025) have intensified droughts, contributing to a significant transition in the contemporary fire regime. This alteration is characterized by increased total burned area, larger mean fire size, and an expanded fire season. With the aim of providing the first robust set of evidence on this structural transition, this study has the following objectives: (i) to quantify and test the significance of long‐term temporal trends in total burned area (BA) and mean fire size (MFS); (ii) to model the temporal MFS and BA dynamics, determining the relative importance of the influence of climatic, hydrological (flood pulse), and fuel factors; and (iii) to detect and quantify the magnitude of changes in fire seasonality metrics (onset, peak, cessation, and duration) and their respective main drivers. We aim to assess whether the biome shows evidence of a contemporary transition in fire regime characteristics, and to evaluate changes relative to hydrological and climatic drivers. Our goal for this study is to provide a robust scientific basis to inform multi‐scalar governance policies aimed at the Pantanal and other tropical wetlands.

## 2 Methodology

### 2.1 Study Area

**FIGURE 1:**
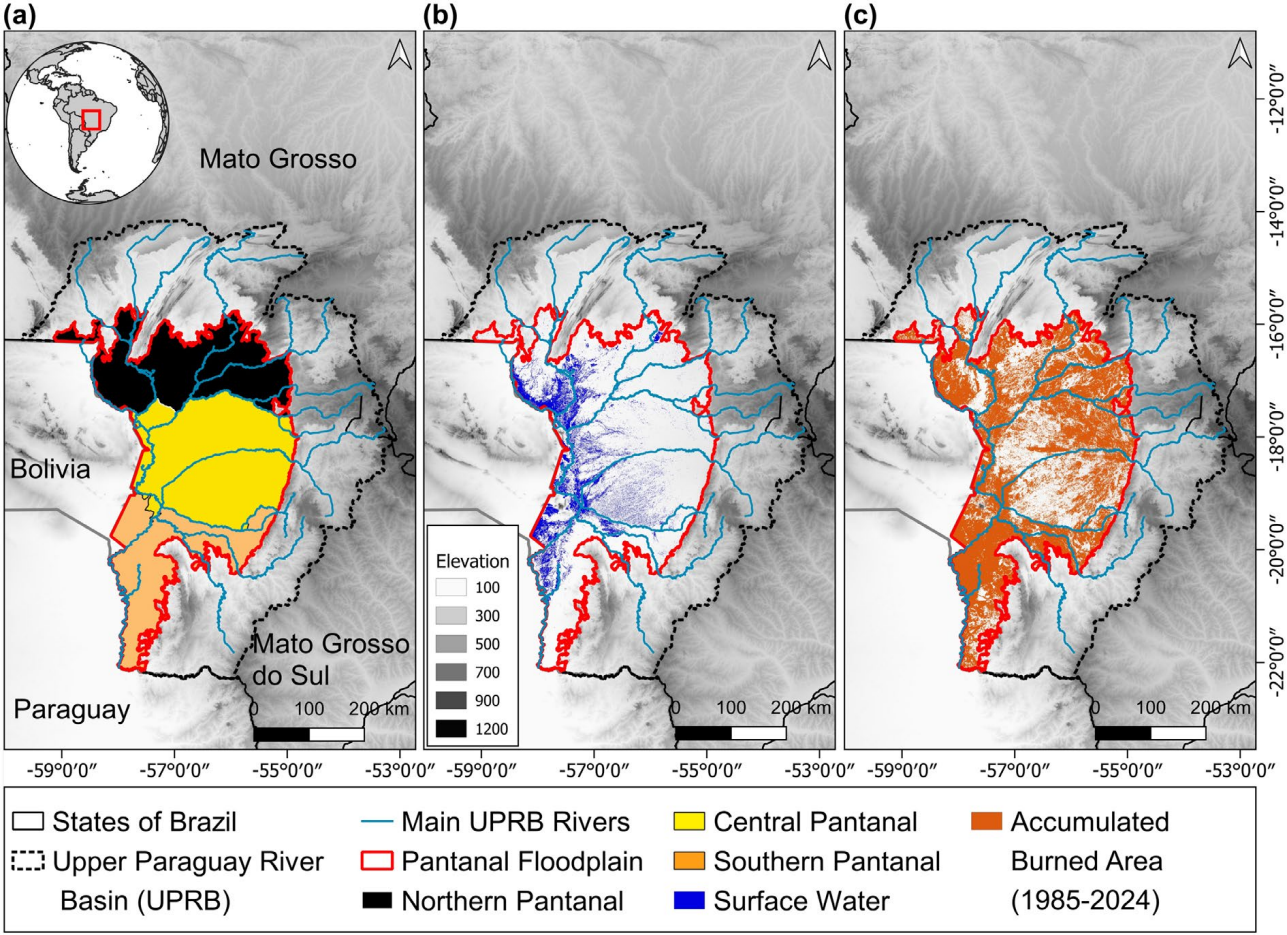
Location of the study area and spatial distribution of surface water and accumulated burned area in the Pantanal. Panel (a) illustrates the location of the Pantanal and the division of the study area into three sub‐regions (Northern, Central, and Southern Pantanal) used for analysis, based on Silva and Abdon (1998). Panel (b) shows the surface water area referring to the year 1985, marking the beginning of the evaluated time series. Panel (c) highlights the total burned area recorded in the Pantanal throughout the entire study period (1985–2024). The main hydrography and the surrounding elevated Planalto plateau areas, which drain into the alluvial plain, are also indicated. Map lines delineate study areas and do not necessarily depict accepted national boundaries.

The Pantanal is the largest continuous alluvial floodplain in the world, located in the Upper Paraguay River Basin (UPRB) in South America, and seasonally flooded by rivers originating in the higher elevation portion of the basin known as the *Planalto* (Figure 1). The study focuses on the Brazilian portion of the Pantanal, where ~75% of this wetland is located (Tomas et al. 2019). The Pantanal is characterized by an extremely flat topography, with a subtle hydrological gradient that gently slopes from north to south. The relief, combined with geomorphological bottlenecks, constrains the seasonal flood pulse, the primary determinant of the landscape (Junk and Nunes da Cunha 2016; Stevaux et al. 2020). Consequently, overbank flows from the Paraguay River and its tributaries linger across the plains due to restricted drainage (Assine et al. 2015). Within the Brazilian territory, fire management is regulated by federal and state‐level policies, including the National Policy for Integrated Fire Management and the recently enacted Pantanal Law, which establish guidelines for fire prevention, prescribed burning, integrated fire management, and biome conservation (Brazil 2024, 2025).

The climate is classified as Tropical Savanna (Aw, according to the Koppen‐Geiger classification), with rainy summers and dry winters, featuring an annual precipitation ranging from 800 to 1300 mm (Kottek et al. 2006; Bergier 2013). The flood pulse is a result of the austral summer rains, modulated mainly by the Intertropical Convergence Zone and the South Atlantic Convergence Zone, mechanisms that drive the critical moisture transport originating from the Amazon Forest, and which generate interannual variations in the extent of the flooded area (Oliveira Vieira et al. 2013; Bergier et al. 2018; Pereira et al. 2021). Due to the slow displacement of the flood pulse, which can take up to 3 months to reach the south of the floodplain (Stevaux et al. 2020), the maximum flooding in the southern region often coincides with the onset of the dry season, creating distinct regional flammability patterns (Arruda et al. 2016).

The vegetation is a complex mosaic of physiognomies (Ab'Saber 1988; Pott and da Silva 2015), being a contact area among species from the Cerrado, Amazon, Atlantic Forest, and Chaco biomes. This floristic diversity is sustained by the water and edaphic variation, encompassing savannas, flooded and non‐flooded grasslands, semi‐deciduous forests, gallery forests, and *cerradões* (dense savanna formations) (Tomas et al. 2019). While the spatial distribution of these fuel types is directly controlled by flooding depth and duration (Silva et al. 2000), their phenological cycles are driven by a complex interaction between the flood pulse, local precipitation, and temperature (Aoki et al. 2021).

Given the environmental heterogeneity and the latitudinal gradient of the flood pulse, we also analyzed the Pantanal by sub‐regions. We adopted the official territorial limit of the Pantanal biome defined by the Brazilian Institute of Geography and Statistics (IBGE‐Instituto Brasileiro de Geografia e Estatística 2025). Within this boundary, we grouped the 11 physiographic sub‐regions defined by Silva and Abdon (1998) into three broader sectors: Northern, Central, and Southern Pantanal (Figure 1). This grouping follows the main north–south hydrological gradient of the flood pulse, with earlier flooding in the northern floodplain and delayed flooding toward the southern Pantanal, while also preserving broad differences in vegetation, soil, and floodplain structure among sub‐regions (Hamilton et al. 1996; Silva et al. 2000; Stevaux et al. 2020; Marengo et al. 2021). This approach allowed us to compare fire dynamics under distinct hydrological contexts without overfragmenting the analysis into smaller sub‐regions. The grouped sub‐regions are: Northern Pantanal (Cáceres, Poconé, and Barão de Melgaço); Central Pantanal (Paraguai, Paiaguás, and Nhecolândia); and Southern Pantanal (Nabileque, Porto Murtinho, Miranda, Aquidauana, and Abobral) (Figure 1 and Table S1).

### 2.2 Data Acquisition and Pre‐Processing

The analysis of the fire regime was based on a 40‐year time series (1985–2024) covering the Pantanal biome and its sub‐regions. All variables were originally obtained from monthly rasters and spatially aggregated by the regional mean over the biome and sub‐regions, ensuring the representativeness of the region's hydrological, climatic, and fuel condition dynamics. We also aggregated the monthly data at an annual scale. The list of predictor variables used, and their references is presented in Table 1. Predictors were selected based on literature supporting their direct influence on fire dynamics, focusing on continuous hydroclimatic variables and hazard indices that modulate fuel moisture and fire propagation at the regional scale (Cawson et al. 2024; Vasconcelos et al. 2025). All selected predictors represent spatially consistent, monthly variables available for the entire 1985–2024 period across the Pantanal biome and its sub‐regions.

**TABLE 1:** Summary of variables used in fire regime modeling and trend analyses.

Source	Variable	Unit	References
ERA5 (ECMWF/Copernicus)	Evapotranspiration	mm	Hersbach et al. 2020
	Precipitation	mm	Hersbach et al. 2020
	2 m temperature	°C	Hersbach et al. 2020
	10 m wind gust	m s^−1^	Hersbach et al. 2020
	Convective available potential energy (CAPE)	J Kg^−1^	Hersbach et al. 2020
CEMS (ECMWF/Copernicus)	Buildup index (BUI)	—	Vitolo et al. 2020
MapBiomas	Burned area (BA)	ha	Alencar et al. 2022
	Surface water	ha	Souza Jr et al. 2020
Derived (ERA5)	Vapor pressure deficit (VPD)	kPa	Tetens 1930
	Standardized precipitation index (SPI)	—	McKee et al. 1993
Derived (MapBiomas)	Mean fire size (MFS)	ha	García et al. 2022

*Note:* Description, data sources, and units of the fire, climatic, fuel condition, and hydrological data used.

The response variable burned area (BA) was sourced from the MapBiomas Fire dataset, chosen for its 40‐year temporal coverage and 30 m resolution mapping based on Landsat imagery and machine‐learning classification (Alencar et al. 2022). Validation analyses report an overall accuracy of approximately 89%, with slightly higher omission than commission errors, indicating conservative burned area estimates and partial divergence with other global burned area products due to differences in spatial resolution and detection sensitivity (Alencar et al. 2022). The hydrological predictor surface water was extracted from the MapBiomas Water dataset, which is derived from its Land Use and Land Cover (LULC) mapping (Souza Jr et al. 2020). This dataset also relies on the Landsat series and machine learning methods, ensuring spatial and temporal consistency with the fire data.

Climatic variables were obtained from the ERA5 reanalysis (Hersbach et al. 2020). ERA5 is a reanalysis product from ECMWF that merges modeled data with observational inputs every 12 h to provide precise estimates of the global atmospheric state. We selected ERA5 due to its extended temporal coverage (1940‐present), global consistency, and previous use in hydroclimatic and fire‐related studies in the Pantanal and Upper Paraguay River Basin (Hersbach et al. 2020; Correa et al. 2022; Caballero et al. 2025; Belém et al. 2026). The Build Up Index (BUI), a key component of the Canadian Fire Weather Index system (FWI) (Vitolo et al. 2020), was included as a proxy for the potential availability of fuel for combustion. The BUI integrates medium and long‐term fuel moisture conditions and is therefore more directly related to combustible material accumulation and dryness. Other component variables from the FWI were not used to avoid redundancy with climatic predictors already included in the models and to preserve the interpretability of individual drivers.

The dataset also included three variables calculated from primary data: the fire metric mean fire size (MFS), the vapor pressure deficit (VPD), and the standardized precipitation index (SPI). The MFS was determined annually by dividing the total burned area (in hectares) by the total number of individual fire scars, identified as connected burned area pixels from MapBiomas data (1985–2024). This metric reflects structural fire‐regime properties and fire‐scar extent rather than total affected area alone (Archibald et al. 2013; García et al. 2022). Although monthly burned area data is available, MFS was calculated annually to preserve the 40‐year temporal coverage and ensure that multi‐week fire activity spanning calendar month boundaries during the dry season is captured as continuous spatial units. Furthermore, annual aggregation is ecologically sound for the Pantanal, as the wet season occurring between the end of one year and the beginning of the next extinguishes fires and prevents scar aggregation from crossing calendar years. Thus, annual MFS serves as a robust structural proxy for average fire scar size across long temporal series.

To estimate VPD, an important proxy for atmospheric dryness and strongly related to wildfire activity (Barros‐Rosa et al. 2022; Cawson et al. 2024), we used temperature at 2 m (T) and dew point temperature (Td) to estimate saturated vapor pressure (es (Equation 1)) and actual vapor pressure (ea (Equation 2)) (Tetens 1930). As vapor pressure deficit increases, the amount of moisture in vegetation and soil decreases, leading to drier conditions. These variables were then used to obtain the VPD (in hPa) (Equation 3):

(1) $$\mathrm{es}=6.11\times 10^{\left(\frac{7.5\times T}{237.3+T}\right)}$$

(2) $$\mathrm{ea}=6.11\times 10^{\left(\frac{7.5\times \mathrm{Td}}{237.3+\mathrm{Td}}\right)}$$

(3) VPD=es–ea

SPI was calculated from monthly precipitation data to represent the drought in the Pantanal. The purpose of this index is to measure rainfall deficit on different time scales (McKee et al. 1993). We calculated the SPI for different time lags: 1 month (SPI‐1); 3 months (SPI‐3); 6 months (SPI‐6); and 12 months (SPI‐12) (Spinoni et al. 2014, 2015).

For time series analysis, autoregressive predictors (BA and MFS lags) were included in all models to control for temporal inertia and residual autocorrelation of the response variable (Khodaee et al. 2024). Overall, autoregressive predictors demonstrate a high contribution in predictive models, but the discussion and graphical presentation will focus exclusively on the main biophysical predictors, allowing the causal influence of the environment on fire dynamics to be isolated. We used lags of 1, 3, 6, and 12 months for the monthly BA models and 1, 2, and 3 years for the annual MFS models (Khodaee et al. 2024). Additionally, deviation variables were calculated over 1, 2, and 3‐year windows. These deviation variables represent the average of the differences between the monthly value and the historical mean of the respective month, allowing for the assessment of the long‐term anomaly of each predictor (Khodaee et al. 2024). The same lags and deviations were produced for all predictors, except for the SPI, which is already a time‐scale‐based metric.

### 2.3 Temporal Trend Analysis

The first analytical step focused on quantifying the temporal trends of the response variables (BA and MFS). The annual time series of both variables was examined to detect monotonic trends and long‐term structural breaks. To identify the presence and location of any Change Points in the time series, Segmented Regression analysis (Muggeo 2003) was applied. This method allows the linear model to be fitted in segments, providing a visual and statistical basis for identifying abrupt changes in the time series. For BA, we also checked the trend in monthly data.

The statistical significance of any monotonic trend in the series was assessed using the Modified Mann‐Kendall Test, a non‐parametric test robust to non‐normality and the presence of outliers (Hamed and Rao 1998). The magnitude of the trend was quantified by the Sen's Slope Estimator, chosen for its robustness to data distribution (Sen 1968). Additionally, the non‐parametric smoothing method LOESS (Locally Estimated Scatterplot Smoothing) was applied to identify and visualize long‐term non‐linear patterns in the time series without statistical significance for a trend (Cleveland 1979).

### 2.4 Data Analysis and Predictive Modeling

The modeling of BA and MFS dynamics was conducted with the objective of identifying the main predictors of fire events in the Pantanal. Firstly, simple univariate models (linear and exponential) were constructed to test the individual relationship of each predictor with the burned area and validate the theoretical premises of known causal relationships. Subsequently, the elastic net (EN) algorithm, a regularized regression Machine Learning model, was selected as the reference model. EN was selected due to its ability to offer a balance between predictive capacity and the interpretability of the coefficients (Zou and Hastie 2005). This is achieved through the combination of Lasso (L1) penalties for feature selection and Ridge (L2) penalties to mitigate overfitting (Zou and Hastie 2005). The choice of EN was preferred over more complex tree‐based models, especially for the annual MFS series, due to the limited number of observations (*N* = 40), which weakens the robustness of methods that require a large volume of data. For monthly BA, four models were constructed: one using filtered data for the entire Pantanal, and the other three using filtered data by sub‐region (Northern, Central, and Southern Pantanal). The MFS modeling was conducted exclusively at the scale of the Pantanal biome as a whole, because the metric is highly sensitive to spatial delimitation, and large fires could cross sub‐regional divisions (mainly delimited by rivers), which would distort its real magnitude. In contrast, the probability of spread to the surrounding plateau (Planalto) is low, validating the analysis across the entire Pantanal.

#### 2.4.1 Variable Selection and Pre‐Processing

The treatment of response variables was tailored to the requirements of each modeling approach. For the monthly models, burned area (BA) was log‐transformed (log_10_) to stabilize variance and ensure that model residuals approximated a normal distribution (Box and Cox 1964). In contrast, mean fire size (MFS) for the annual models exhibited high right‐skewness that could not be corrected through transformation to meet Gaussian assumptions. Consequently, we modeled MFS in its original scale using a Tweedie distribution, which is specifically suited for continuous, non‐negative data with high skewness (Dunn and Smyth 2005).

We applied an initial data filtering through the Mann–Whitney *U* Test (MacFarland and Yates 2016; Khodaee et al. 2024). For this, BA and MFS data were discretized into “low” (25th Percentile) and “high” (95th Percentile) classes, and only variables that showed a statistically significant difference (*p* < 0.01) in the distribution between these extremes were retained for the modeling phase. This step ensured that only predictors with fundamental discriminatory power were included.

To mitigate the inherent imbalance in time series of extreme fire events, a weighting scheme was applied exclusively to the monthly data (BA) (He and Ma 2013; Jain et al. 2020). This scheme uses a cubic penalty function, (BA/max (BA))^3^, to drastically prioritize the minimization of error in the upper tail of the distribution. The multiplier factor was calibrated to confer a weight of 10 (ten times greater than the minimum weight) to the maximum events, directing the model to focus on the prediction of extreme events. Such a scheme was not applied to the annual models, due to the lower number of observations and the inherent robustness of the Tweedie loss function used for annual data.

Although the elastic net is designed to handle correlated predictors, reducing multicollinearity is recommended to improve model parsimony and the stability of coefficient interpretation (Dormann et al. 2013). For this, we used the Variance Inflation Factor (VIF) before modeling BA and MFS. VIF is essential for linear models, as it ensures that the regression coefficients are stable (Chatterjee and Hadi 2015). The process started with the variables that passed the Mann–Whitney *U* Test. In each iteration, we calculated the VIF for each predictor in the linear model, and the variable with the highest VIF was removed, provided its VIF exceeded the established threshold of 10 (Hair et al. 2013). This procedure was repeated until all remaining variables presented a VIF below the threshold, ensuring the stability of the regression coefficients.

#### 2.4.2 Predictive Model Fitting

The cost functions were defined in alignment with the distribution of the response variable. For the transformed monthly variable (log_10_‐transformed BA), the elastic net was optimized using the squared error loss function, the standard for regression with the assumption of normal residuals. In contrast, for the annual MFS series, due to its non‐normalized distribution, with asymmetry and zeros, the Tweedie loss function (Dunn and Smyth 2005) was employed. Hyperparameter optimization (*α* and *λ*) was performed for both models using a grid search within the validation scheme (Bergstra and Bengio 2012).

The validation strategy was determined by the extent and resolution of the time series. To validate the predictive capacity in future horizons of the monthly BA model, and to mitigate the risk of temporal data leakage in a long series, time series cross‐validation (CV), based on a fixed window rolling forecast (Shojaei and Flood 2018), was employed. This scheme simulates the operational use of the model by ensuring that test data are always chronologically future relative to the training data. The process was implemented with a five‐fold sliding window. In each fold, 80% of the data was used for training and 20% for validation, with the constraint that the minimum test period be 12 months to assess prediction on annual and seasonally complete scales. For the annual model (MFS), due to the limited number of observations, which makes the use of cross‐validation unfeasible, we adopted the temporal hold‐out split (80/20) (Kuhn and Johnson 2013) for the final evaluation. The split was performed chronologically, dedicating 80% of the time series for model fitting and tuning and 20% most recent years as a test set unseen by the model during training.

#### 2.4.3 Performance Evaluation and Variable Importance

Elastic net model performance was quantified using the coefficient of determination (*R* ^2^) and root mean square error (RMSE), calculated according to the respective validation schemes: Temporal CV for monthly BA, and Hold‐Out for annual MFS. *R* ^2^ assesses the proportion of variance explained and RMSE provides the mean error on the scale of the response variable (log_10_ ha for BA and ha for MFS). Variable importance was determined by extracting the absolute value of the regression coefficient of each predictor in the elastic net model. For the monthly BA model, both performance metrics and variable importance were calculated per temporal CV fold and then aggregated by the mean to represent overall performance and predictive weight. In contrast, for the annual MFS model, variable importance was obtained in a single final evaluation on the single test set. The final mean importances guided the discussion on the fire control mechanisms in the Pantanal.

### 2.5 Seasonality Change Analysis

To investigate whether the seasonal fire regime and its main environmental drivers underwent significant alterations over the study period, trend analyses were conducted on seasonality metrics produced from BA data and its main drivers. Given the latitudinal seasonal variability in the Pantanal, this analysis was restricted to the sub‐regions, unlike the previous analyses that were also conducted at the scale of the entire biome.

#### 2.5.1 Definition of the Hydrological Year and Seasonal Metrics

The temporal dynamics of fire in the Pantanal are intrinsically linked to seasonal climatic and flood cycles, which often overlap the traditional calendar year (January to December) and vary between sub‐regions due to the latitudinal extent of the biome. To overcome the limitation of a fixed seasonal year and ensure that the complete annual cycle (accumulation, peak, and decline) of each phenomenon is fully captured, the analysis was framed within the concept of the dynamic seasonal year (Luković et al. 2021). This 12‐month year is defined individually for each variable and sub‐region, starting in the month immediately preceding the month in which the historical minimum monthly mean value is recorded (historical climatology). This delimitation is crucial because it establishes a stable reference and allows seasonality metrics to be calculated based on the total accumulated sum of the annual cycle, rather than on an artificially divided cycle.

The seasonality metrics ‘onset’, ‘cessation’, and ‘duration’ were calculated annually through the cumulative percentile method (Luković et al. 2021), which identifies inflection points in the dynamic seasonal year. The onset marks the month when the accumulated sum reaches the lower percentile (start of the high phase of the phenomenon), and cessation marks the month that reaches the upper percentile (start of the low phase). We used the thresholds of 25% for ‘onset’ and 75% for ‘cessation’. ‘duration’ was determined as the period between these two inflection points in the series.

However, exclusively for surface water, the duration was determined by the total count of months in the seasonal cycle in which the surface water value was above the historical median (50th percentile) of the respective sub‐region. This methodological choice allows for a more robust quantification of the effective time of high‐level flooding. We also used the metric ‘peak’ to define the month of highest values of the variables.

#### 2.5.2 Detection and Magnitude of Alteration Trends

Each seasonal metric was treated as a time series and assessed using long‐term trend tests. For the detection of significant trends in regime alteration over time, we used the Modified Mann‐Kendall non‐parametric test (Hamed and Rao 1998). The magnitude of each detected trend was determined using Sen's slope, quantifying the rate of advance or retreat of the phenomenon (e.g., months/year) over the study period.

## 3 Results

### 3.1 Long‐Term Trends in Burned Area and Mean Fire Size

**FIGURE 2:**
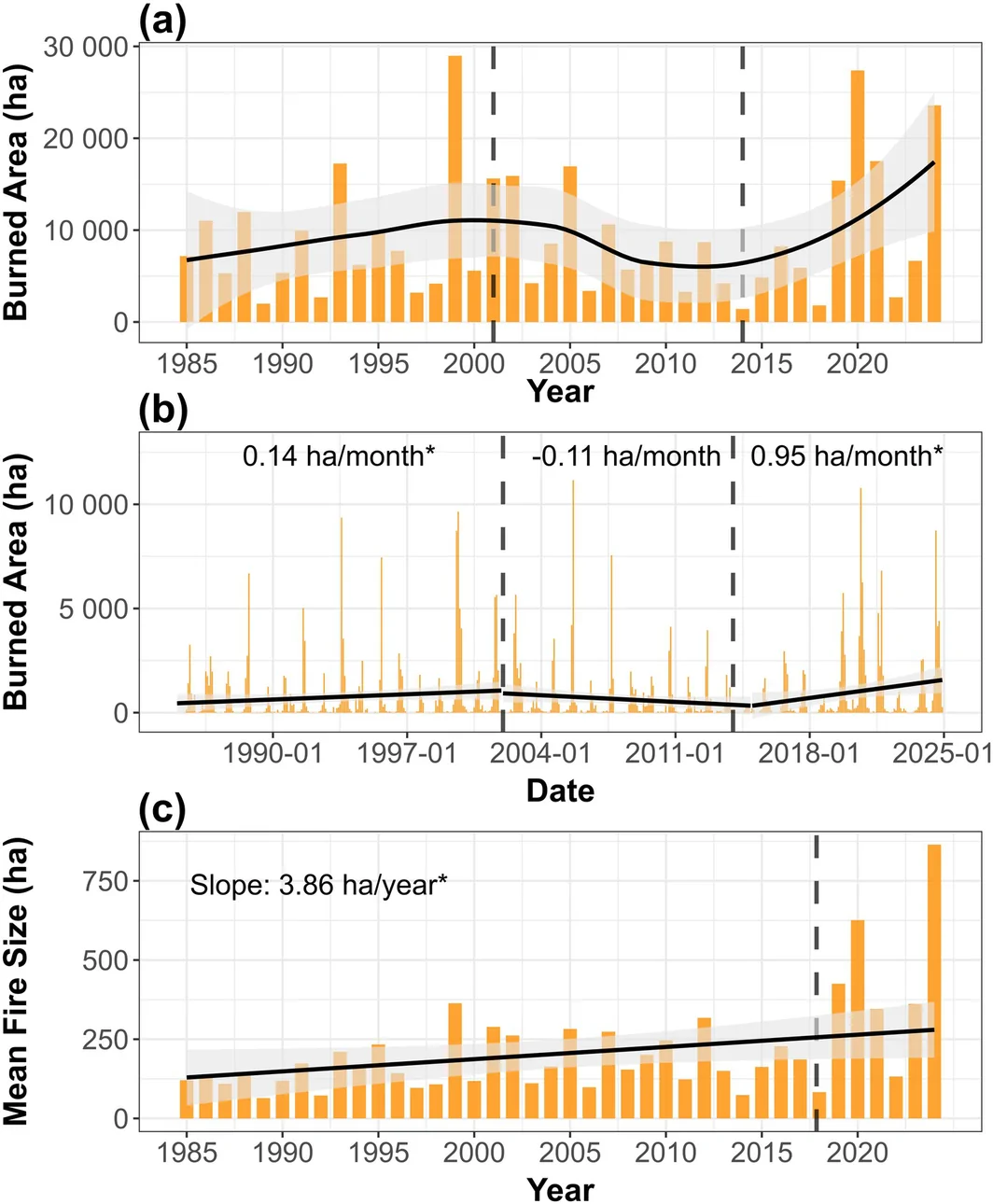
Trends and change points for burned area (BA) and mean fire size (MFS) in the Pantanal (1985–2024). Panels (a) and (b) show the observed values (orange bars) of BA on annual and monthly scales, respectively. Panel (c) represents the annual MFS time series. The vertical dashed lines mark the detected significant change points. The solid black line represents the trend, with the gray area indicating the 95% confidence interval. The slope of significant trends (*p* < 0.05) is identified by a ‘*’. The annual BA trend was not significant and was therefore modeled by LOESS, to demonstrate the potential non‐linear fluctuations in the series.

Our results reveal structural changes in the Pantanal's contemporary fire regime. The segmented regression test identified two change points in the Pantanal BA series (*p* < 0.05): one in 2001 and another in 2014 (Figure 2a). Upon evaluating the annual BA series, we found no significant trends. However, using the monthly data, we were able to identify an increase between 1985 and 2001 (0.14 ha/month), a period of stabilization between 2002 and 2014, and an even more pronounced significant increase starting in 2015 (0.95 ha/month) (Figure 2b). Similar behavior was observed when evaluating BA trends by sub‐regions (Figures S1–S3). For the annual MFS, we identified a linear trend for the entire study period (Figure 2c), with an average annual increase of 3.86 ha/year. Furthermore, we identified a change point in 2018 (Figure 2c), which marks the accentuation of the increasing trend. This indicates that individual fires in the Pantanal have been reaching even greater extents since 1985, and the speed of this increase has intensified further since 2018.

### 3.2 Fire Control Mechanisms (Predictive Modeling)

**FIGURE 3:**
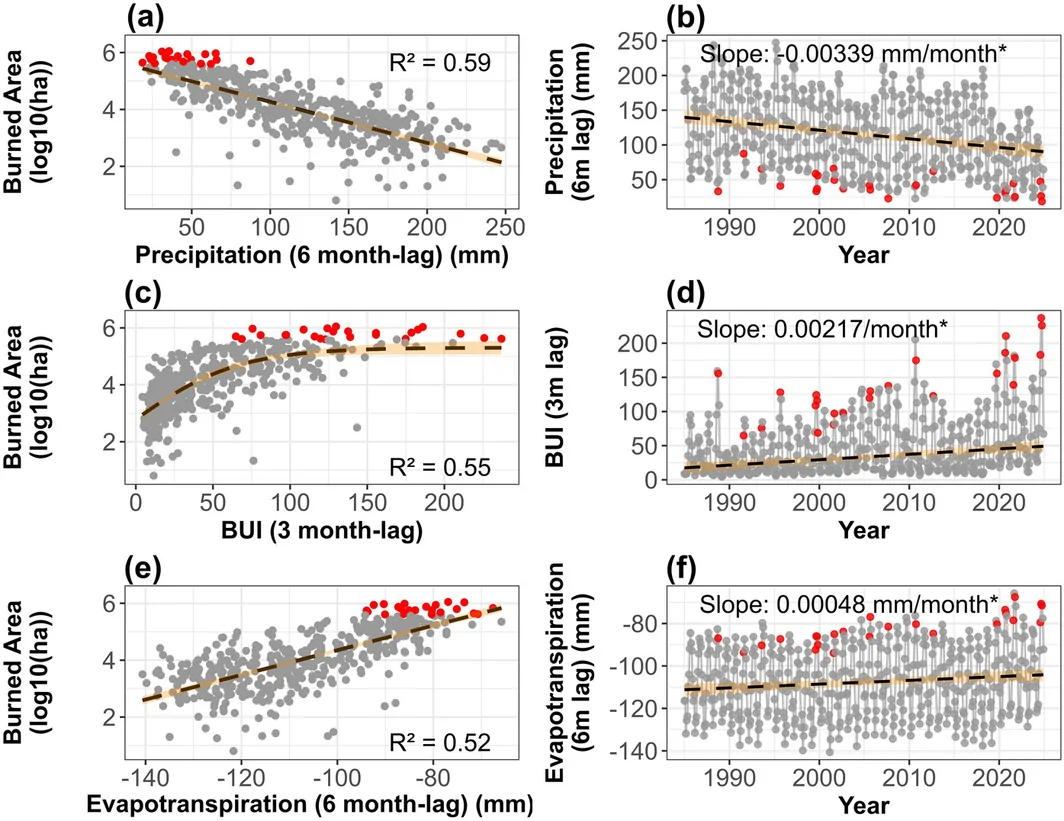
Simple regression of each predictor with monthly burned area (BA) and their temporal trends. Individual predictive relationship and the temporal evolution of the three biophysical predictors with the highest coefficient of determination (*R* ^2^) in relation to monthly BA (1985–2024). The first column panels (a, c, e) display the results of the simple regression: The dashed lines represent the fitted models, and the *R* ^2^ value quantifies the predictive power. The red dots highlight the months of fires classified as extreme (95th Percentile). The second column panels (b, d, f) show the temporal evolution of each predictor, where the superimposed dashed line indicates the long‐term trend. The slope of significant trends (*p* < 0.05) is identified by ‘*’. The orange band indicates the 95% confidence interval.

Upon evaluating the individual relationships between BA and each predictor using simple regressions (linear and exponential), we identified precipitation (6‐month lag), Build Up Index (BUI) (3‐month lag), and Evapotranspiration (6‐month lag) as the variables with the greatest individual influence on BA (Figure 3 and Table S2). Lower precipitation values 6 months prior and higher BUI values 3 months prior show a strong relationship with higher monthly burned area. A reduction in evapotranspiration (values closer to zero) also resulted in increased monthly burned area. All these predictors showed a significant trend in the time series (Figure 3).

The elastic net model for monthly BA demonstrated satisfactory predictive performance over future horizons, with a mean R^2^ of 0.67 and a mean RMSE of 0.5 (log_10_ ha) for the test sets, and with mean test performance comparable to training performance (Table 2). The results show that the model explains slightly more than two‐thirds of the variance in monthly BA, confirming the validity of the elastic net model for predicting monthly BA dynamics.

**TABLE 2:** Cross‐validation and temporal partitioning metrics for the monthly burned area elastic net model in the Pantanal.

Fold	Train period	Test period	Train *R* ^2^	Test *R* ^2^	Train RMSE	Test RMSE
1	1985–2000	2000–2004	0.71	0.77	0.57	0.43
2	1989–2005	2005–2010	0.70	0.36	0.56	0.83
3	1994–2011	2011–2016	0.68	0.85	0.58	0.31
4	1999–2016	2017–2020	0.70	0.76	0.51	0.43
5	2004–2021	2021–2024	0.64	0.60	0.57	0.58
Mean	—	—	0.69	0.67	0.56	0.52

*Note:* The table details the training and test partitions used in each of the five folds time windows for the evaluation of the model's temporal stability.

**FIGURE 4:**
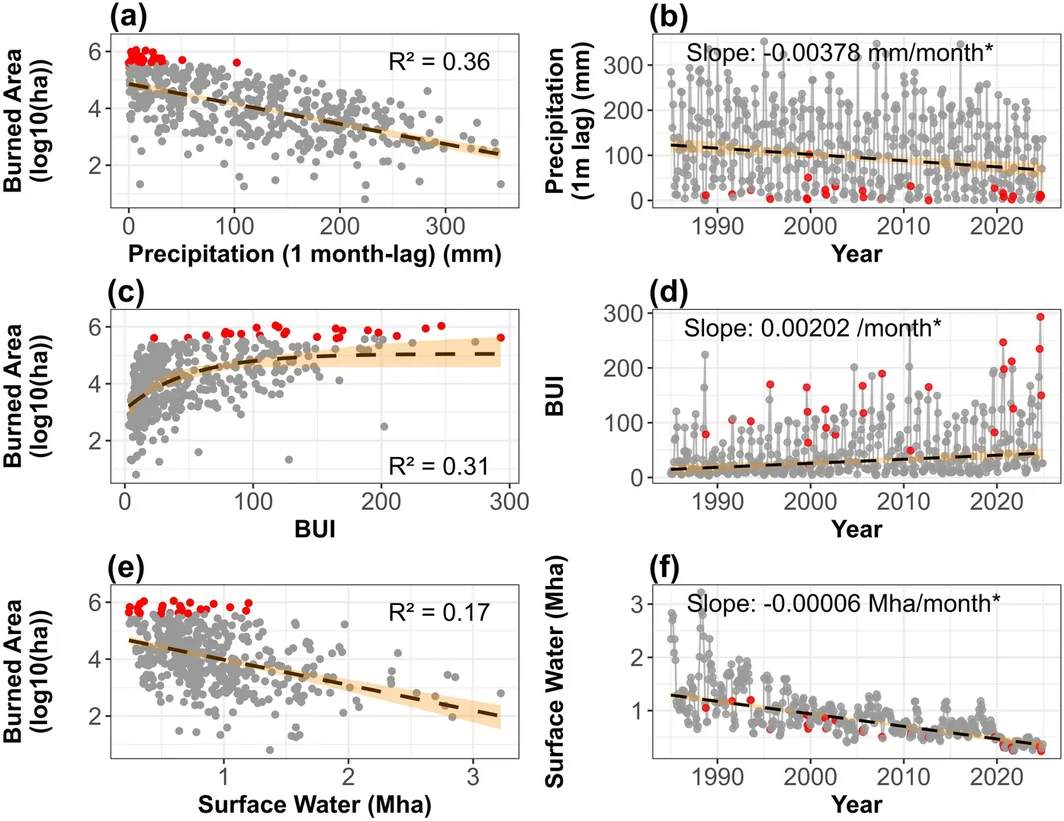
Relationships and monthly temporal trends of predictors selected by the elastic net model for burned area (BA) in the Pantanal (1985–2024). The figure displays the factors of highest relative importance for the modeling of monthly BA (Panels a, c, e), after the removal of collinearity by the Variance Inflation Factor VIF, and their respective trend time series (Panels b, d, f). The left column relates the monthly BA, highlighting extreme events (red dots; 95th Percentile), with each of the main predictors, using linear or exponential regression (in the case of BUI). The right column illustrates the monthly time series of each predictor, where the dashed line indicates the long‐term trend. The slope of significant trends (*p* < 0.05) is identified by ‘*’. The dashed orange band indicates the 95% confidence interval.

The hierarchy of importance of the predictor variables for the monthly BA model in the Pantanal is led by precipitation (1‐Month Lag), buildup index (BUI), and surface water (Table 3 and Table S3). Generally, lower precipitation and surface water values result in an increase in burned area, while an increase in BUI is positively related to burned area (Figure 4). All these variables also showed a significant linear trend.

**TABLE 3:** Relative importance of the most important predictors in the elastic net model for monthly burned area and annual mean fire size.

Response variable	Predictor variable	Importance
Burned area	Burned Area (6 months lag)	0.29
	Precipitation (1 month lag)	0.28
	Buildup Index	0.20
	Surface Water	0.12
Mean fire size	Buildup Index	0.58
	CAPE	0.43
	Surface Water	0.23
	Evapotranspiration	0.22

*Note:* The importance of each variable was estimated from the absolute values of the standardized regression coefficients of the elastic net model. This value reflects the strength of each predictor's contribution to the variance of the response variable.

The sub‐regional models demonstrated a variable but robust predictive capacity for monthly BA. The highest test performance, evidencing the model's best generalization capacity, was observed in the Northern Pantanal, with a mean *R* ^2^ of 0.86 and an RMSE of 0.4 (log_10_ ha). The Central Pantanal model retained reasonable explanatory power, with a mean *R* ^2^ of 0.56 and an RMSE of 0.64 (log_10_ ha). The model for the Southern Pantanal achieved intermediate performance, with a mean *R* ^2^ of 0.62 and a mean RMSE of 0.63 (log_10_ ha). The evaluation using temporal forward cross‐validation demonstrated that the predictive performance of the sub‐regional models was consistent across folds (Tables S4–S6). However, similar to the general model, folds 2 and 5, corresponding to the periods 2005–2010 and 2021–2024, showed the lowest performance. This pattern suggests greater difficulty in capturing the variance of monthly burned area during these intervals, indicating the presence of additional sources of variability not fully represented in the model.

Regarding the predictors, there was little difference between the sub‐regional models and the model considering the entire biome (Tables S7–S9). The highlight is VPD (vapor pressure deficit), which was the most important biophysical predictor in all sub‐regions. This is a notable result, given that VPD was not selected for the general Pantanal model after the application of VIF due to its high collinearity with other predictors, such as BUI (Figure S4). However, VPD showed a high individual relationship with BA (Figure S4), justifying its inclusion and prominence in the sub‐regional models.

**FIGURE 5:**
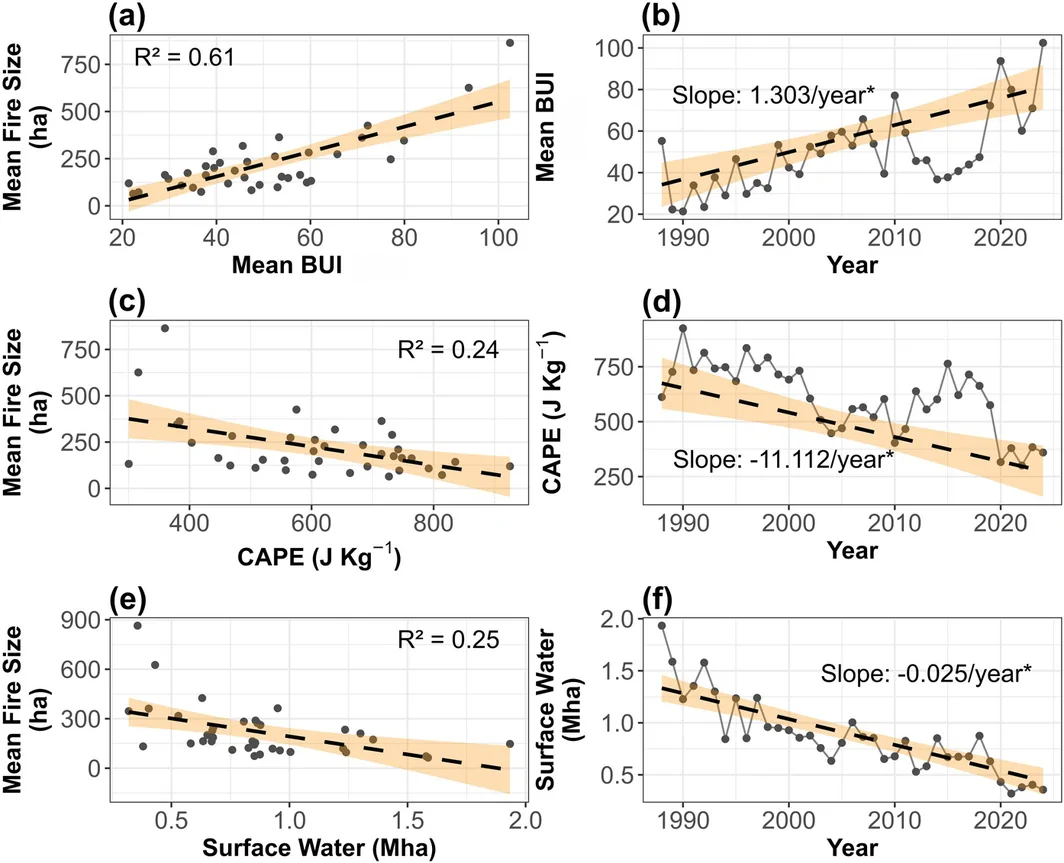
Relationships and annual temporal trends of predictors selected by the elastic net model for mean fire size (MFS; ha) in the Pantanal (1985–2024): Buildup index, a dimensionless index; convective available potential energy (CAPE; J kg^−1^); and surface water (Mha). The figure displays the factors of highest relative importance for the modeling of annual MFS (Panels a, c, e), after the removal of collinearity by the Variance Inflation Factor (VIF), and their respective trend time series (Panels b, d, f). The left column relates the annual MFS with each predictor, using linear regression. The right column illustrates the annual time series of each predictor, where the dashed line indicates the long‐term trend. The slope of significant trends (*p* < 0.05) is identified by ‘*’. The dashed orange band indicates the 95% confidence interval.

The annual model for MFS (Table 3) also showed satisfactory performance, with an *R* ^2^ of 0.6 for the training set, an *R* ^2^ of 0.8 for the test set, and an RMSE of 51.3 ha for training and 87.7 ha for the test set. The MFS model also validated BUI and surface water as important predictors but introduced convective available potential energy (CAPE) (Figure 5) as a relevant driver. The negative relationship between the annual mean CAPE and MFS, indicating that years of higher CAPE result in smaller fires (due to suppressive convective activity), is complemented by a significant trend of decline in annual CAPE (Figure 5d). This decline suggests a progressive reduction in the hydrological mitigation factor, contributing to the observed increase in MFS. The annual values of BUI and surface water also showed a significant change in the time series.

### 3.3 Regional Heterogeneity and Seasonal Alteration Trends

**FIGURE 6:**
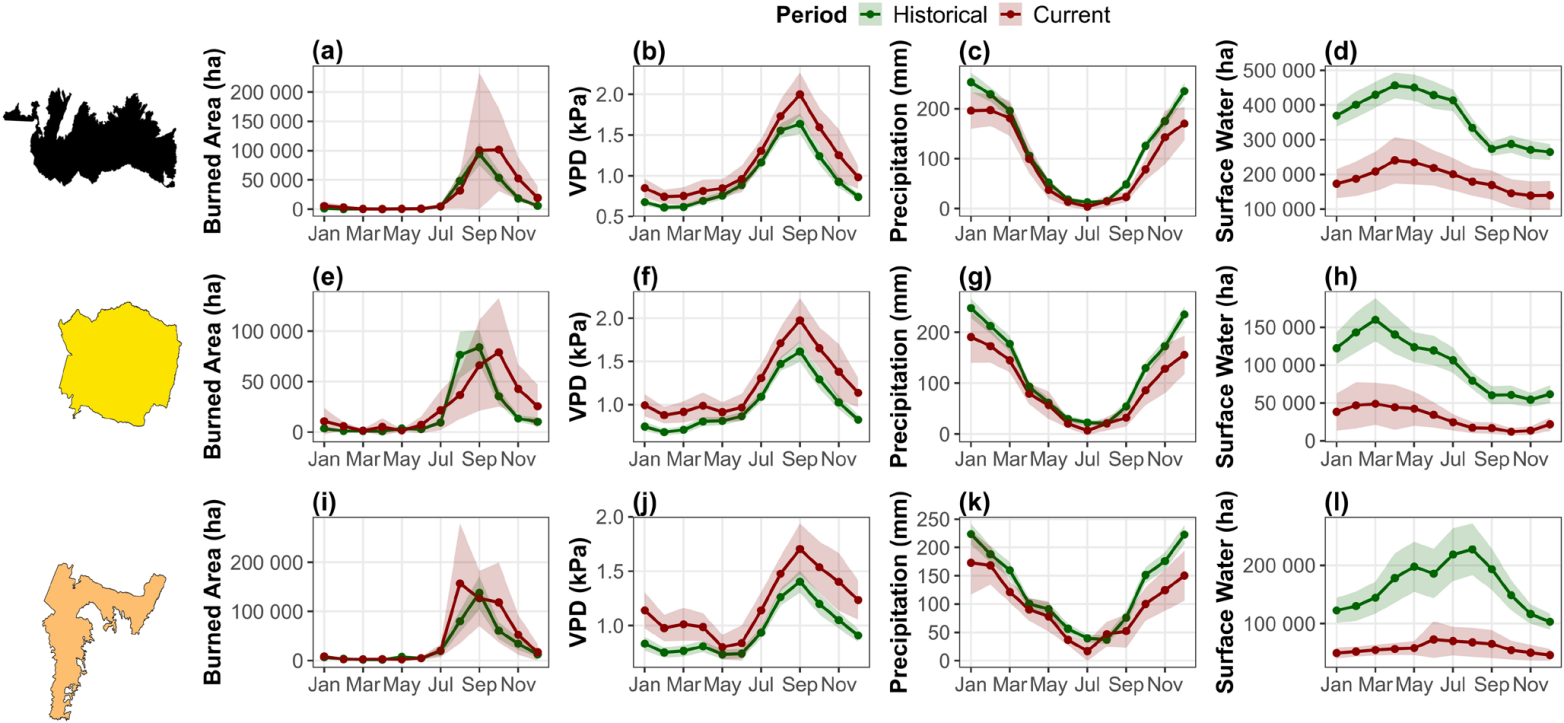
Analysis of the seasonality of fire and its main predictors by Pantanal sub‐region. The graphs compare the monthly means shown by solid lines and the 95% confidence intervals, shown by shaded areas, between the historical period (1985–2014, green color) and the recent period (2015–2024, red color). The columns represent burned area (ha), vapor pressure deficit (VPD; kPa), precipitation (mm), and surface water (ha). The subplot rows divide the analysis by sub‐region: Northern (top row), Central Pantanal (middle row), and Southern Pantanal (bottom row).

The seasonality alteration analysis first quantified the contrast between the historical seasonal curve (1985–2014) and the curve of the recent period (2015–2024) (Figure 6). Although BUI was a key predictor in the general model, it was decided to present VPD in this seasonality analysis. The high collinearity between the variables allows for this substitution; furthermore, VPD offers greater ease of physical interpretation in the atmospheric context and was highlighted in the sub‐regional models. Figure 6 illustrates the deregulation in the Pantanal's hydroclimatic coupling, evidenced by the pronounced displacement of the seasonal curves in all sub‐regions, especially for surface water and burned area. Although VPD showed an increase in the recent period, coinciding with the fire season, no significant trend of alteration in the seasonality of this variable was identified.

Table 4 statistically quantifies these alterations. The most expressive phenomenon is the highly significant trend of reduction in the duration of the flood pulse in all sub‐regions, occurring at an average rate of 0.3 month/year, equivalent to about 10 days/year assuming a 30‐day month. Significant alterations in the fire season were also observed. In the Northern Pantanal, the peak of the fire season, which traditionally occurred in September, is transitioning to October (Figure 6; Table 4), consistent with the significant trend of delay in precipitation onset, moving from November to December. This delay prolongs fuel desiccation. Sen's Slope values very close to 0 (< 0.01) indicate a real but extremely small magnitude of alteration, which is common in discrete variables.

**TABLE 4:** Trends in the seasonality of fire and its main drivers in Pantanal.

Region	Variable	*p*	Sen's slope
Northern Pantanal	Fire season—peak month	0.01	< 0.01
Northern Pantanal	Flood pulse—duration	0.00	−0.34
Northern Pantanal	Precipitation—onset	0.02	< 0.01
Central Pantanal	Fire season—duration	0.03	< 0.01
Central Pantanal	Fire season—cessation	0.002	0.04
Central Pantanal	Flood pulse—duration	0.00	−0.34
Central Pantanal	Flood pulse—cessation	0.04	< 0.01
Southern Pantanal	Flood pulse—duration	0.00	−0.35

*Note:* The table presents the magnitude and direction of the trends identified for the seasonality metrics (onset, cessation, duration, and peak) of fire, flood pulse, and precipitation. The trend was assessed using the Modified Mann‐Kendall Test for statistical significance, and its magnitude is quantified by Sen's Slope (month/year). Only statistically significant results (*p* < 0.05) are included.

For the Central Pantanal, the main change lies in the cessation of the fire season, which is delayed at an average rate of 0.04 month/year (1.2 days/year), shifting the end of the season from September to November over the 1985–2024 period and, consequently, significantly extending the duration of the fire season. Overall, these trends confirm the contraction of the flooding period in all sub‐regions, along with pronounced changes in fire seasonality in the North and Central Pantanal regions, indicating a clear deregulation in the hydroclimatic coupling that historically defined ignition and extinction of fire.

## 4 Discussion

### 4.1 Structural Change in the Pantanal's Fire Regime

The results reveal a critical multi‐decadal transformation of the Pantanal's contemporary fire regime, characterized by increases in burned area (BA), mean fire size (MFS), and fire‐season length over 1985–2024. This transformation appears to have unfolded in two main phases: an initial increase in monthly burned area after 2014 (Figure 2b), followed by a pronounced acceleration in mean fire size after 2018 (Figure 2c). The sequential emergence of these patterns suggests that recent fire‐regime intensification has involved both an expansion of the area affected by fire and a transition toward larger contiguous burned patches. Our modeling framework further indicates that this transition is closely associated with intensified hydroclimatic coupling, particularly through rising vapor pressure deficit (VPD), sustained reductions in precipitation, and shorter flood‐pulse duration. In this context, the systematic decline in surface water and delayed onset of precipitation appear to have contributed to both an expansion and temporal transition in the Pantanal's fire season (Figure 6; Table 4). By extending fuel desiccation and flammability windows, these concurrent hydroclimatic changes reshape historical constraints on fire activity, altering seasonal and spatial dynamics across the world's largest tropical wetland.

By moving beyond the literature's predominant focus on interannual variation of burned area alone (Marques et al. 2021; Kumar et al. 2022; Silva et al. 2022), our findings offer a more comprehensive multi‐decadal assessment of the Pantanal's fire regime. This advance was possible through an integrated modeling approach that combines large‐scale water dynamics (flood pulse) with climatic and fuel condition predictors at high temporal resolution (monthly) over a 40‐year period (1985–2024). By capturing these sub‐annual hydroclimatic interactions (particularly surface water dynamics and atmospheric moisture deficit), our framework complements traditional annual metrics by revealing critical intra‐annual alterations in fire seasonality and size dynamics.

### 4.2 Mechanisms and Drivers of Transition

The structural transition observed in the Pantanal's fire regime is directly linked to the primary climate and hydrological variables selected by our modeling framework—most notably accumulated drought metrics (BUI), surface water extent, precipitation lagged effects, CAPE, and VPD. Although the identified fire regime change can be characterized as anthropogenic in origin (Pausas and Keeley 2014), given the high number of fires at the peak of the dry season when rain episodes are rare, changes in climatic conditions and the lengthening of the fire season create new and dangerous windows for natural ignitions. Specifically, the shift in model drivers demonstrates that fire behavior is no longer constrained solely by peak‐dry season conditions, but rather by the cumulative atmospheric and hydrological deficit established months prior.

In the Central Pantanal, the significant extension of the fire season into late November (Table 4) creates a critical overlap with the onset of the convective season. This onset is marked by elevated CAPE during transitional months, which increases the likelihood of dry‐lightning events. Although the highest frequency of lightning in the Pantanal occurs during November and December (Menezes et al. 2022), these early‐season storms often yield insufficient rainfall to offset accumulated fuel moisture deficits (elevated BUI and VPD). Consequently, lightning strikes occurring under persistent water stress and elevated VPD may increase the risk of natural ignitions before the full establishment of the rainy season, further expanding the fire window. Furthermore, prolonged climatic and water stress increases the vulnerability to self‐ignition in peat soils and those with a high proportion of organic material (Yuan et al. 2021). Such findings are critical in light of Intergovernmental Panel on Climate Change (IPCC) projections, which indicate climate change in the Pantanal by the end of the century could include an increase of up to 7°C, an extension of the dry period, and a 30% reduction in Pantanal rainfall (Marengo et al. 2015, 2016).

The structural alteration of the regional climate and the flood pulse detected in our study, and their associated changes in the contemporary fire regime, gain greater context when confronted with factors external to the Pantanal. Our results indicate that the observed deficits in precipitation and surface water extent align with broader multi‐scalar land‐use and hydrological alterations in the Upper Paraguay River Basin (UPRB). Anthropogenic degradation in the Planalto portion of the Upper Paraguay River Basin has intensified, primarily caused by agricultural expansion, and subsequent reduction of native vegetation, and the increase of water resource use for irrigation and energy (Barros‐Rosa et al. 2025; Nunes et al. 2025). Such practices are, in turn, decreasing the water retention capacity of the entire basin, resulting in a prolonged dry season and greater vulnerability to fire. The contraction of the flood pulse and the subsequent fire regime change observed here serve as a global alert for the extreme vulnerability of other tropical wetlands to hydroclimatic deregulation.

The problem is further amplified by continental‐scale climate teleconnections. Precipitation, which emerged as the primary negative predictor of fire activity in our elastic net models, is largely regulated by atmospheric moisture transport from the Amazon Forest (Bergier et al. 2018). Consequently, deforestation and climatic stress in the Amazon have profound implications for water availability in the Pantanal (Bergier et al. 2018). While some studies suggest that oceanic oscillations (e.g., AMO and ENSO) have a low direct relationship with extreme droughts like those of 2019–2020 (Marengo et al. 2021), alternative evidence highlights the critical role of sea surface temperatures (SST) in both the Pacific and Atlantic oceans as primary drivers of Pantanal rainfall patterns (Thielen et al. 2020). These oceanic indices may act both directly and through a ‘cascade effect’ by modulating Amazonian atmospheric dynamics, which subsequently affects the moisture transport to the UPRB (Bergier et al. 2018; Marengo et al. 2021).

Recent decades have shown an increasing occurrence of both droughts and heatwaves in the Pantanal (Libonati et al. 2022), reflecting broader trends of warming and hydroclimatic variability across tropical South America. Our findings indicate that reduced precipitation, coupled with high atmospheric aridity and surface water contraction, drives the highest burned area and fire size across the 40‐year record. In addition to becoming more frequent, these extremes have increasingly occurred simultaneously, intensifying their combined impacts on the region's ecosystems, as observed in 2020 and 2024 (Libonati et al. 2022; Belém et al. 2026). This growing co‐occurrence of hot and dry conditions creates a feedback that enhances flammability and facilitates the spread of large wildfires.

### 4.3 Ecological and Socioeconomic Impacts

The structural changes in the fire regime, driven by hydroclimatic factors and surface water contraction, threaten to reshape the Pantanal as a socio‐ecological system. When fires reach extreme magnitudes, they become highly detrimental to wetland ecosystems, increasing greenhouse gas emissions through the combustion of peat and organic matter (Page et al. 2002; Van der Werf et al. 2017), while causing long‐lasting ecological impacts, including erosion, nutrient depletion, changes in vegetation structure, and biological invasions (Leeds et al. 2009; Kotze 2013; Balch et al. 2015). Catastrophic fires have also caused massive wildlife mortality, including an estimated 17 million vertebrates killed in 2020 alone (Tomas et al. 2021), threatening ecosystem resilience and service provision. Beyond ecological impacts, prolonged smoke exposure increases respiratory diseases (Lorenz et al. 2024), while recurring fires jeopardize local livelihoods, including jaguar ecotourism (Tortato et al. 2017) and traditional landscape management (Botelho et al. 2025).

Although robust evidence exists regarding these immediate consequences, much less is known about how contemporary transitions in the fire regime, if sustained over time, may reshape the functioning of the Pantanal as a socio‐ecological system. If the transitions identified here persist, impacts are likely to become more frequent and interconnected, generating cascading, non‐linear responses across ecological and social systems. For instance, repeated disruptions of hydrological and fire regimes may compromise migratory species dependent on predictable flood pulses while affecting fisheries and associated livelihoods. Likewise, shifts in fire timing and intensity reduce natural pasture recovery with direct consequences for livestock production, while more frequent wildfires exacerbate population exposure to smoke and public health risks. Rather than producing isolated impacts, interacting climate extremes and altered fire regimes reinforce one another through compound disturbances, progressively reducing ecosystem resilience and increasing the likelihood of shifts toward a new socio‐ecological state characterized by stronger feedbacks among climate extremes, biodiversity loss, ecosystem degradation, and human vulnerability.

### 4.4 Limitations and Future Perspectives

Although our temporal models successfully captured the fire dynamics and their main drivers, recognizing certain methodological limitations remains essential. Despite strong univariate correlations with BA, medium‐term climatic drivers (e.g., precipitation at 6‐month lag) were excluded from the final model due to high collinearity (VIF) and the elastic net L1 penalty. While this yielded more parsimonious models, it constrained the direct interpretability of the isolated climate‐memory effects, requiring long‐term signals to be inferred through immediate hydroclimatic variables. Another limitation is the inability to directly incorporate anthropogenic drivers, such as native vegetation loss and agribusiness expansion. Although these land‐use pressures strongly influence fire dynamics, high‐frequency monthly time series covering the entire 1985–2024 period are currently unavailable at the biome scale, precluding their inclusion in our temporal regression framework. Nevertheless, the reduction in the annual flood pulse serves as a robust proxy for combined climatic stress and land‐use pressures (Barros‐Rosa et al. 2025). From a spatial perspective, while MFS effectively isolates distinct scars, deriving connectivity from annual records may occasionally merge adjacent fires from separate ignitions during extreme dry seasons, potentially overestimating event sizes. Consequently, MFS is best interpreted as an annual structural proxy for fire spread capacity, which could be refined in future studies by coupling high‐frequency active‐fire data (e.g., VIIRS) with spatiotemporal clustering.

Future studies operating at finer spatial scales (e.g., pixel‐based models) should incorporate local terrain attributes, fine‐scale fuel load, and spatial policy enforcement to explore how micro‐environmental heterogeneity interacts with broad hydroclimatic drivers. These fine‐scale attributes could not be integrated into our regional temporal framework because static terrain variables (DEMs) become temporal constants when aggregated monthly, vegetation proxies (NDVI) exhibit severe collinearity with climate metrics, and qualitative policy enforcement cannot be easily quantified over time. Furthermore, accounting for localized enforcement intensity and spatial human activity in future work may help explain the lower predictive performance observed in specific temporal folds (2005–2010 and 2021–2024). Finally, although the four‐decade satellite record represents the most consistent high‐resolution dataset available, it remains a relatively limited window for confirming long‐term regime shifts. These findings should therefore be interpreted as evidence of contemporary changes indicative of a potential transition rather than definitive proof of a new stable state.

### 4.5 Implications for Policies and Multi‐Scalar Governance

Our findings suggest that the ongoing transition in the Pantanal fire regime requires a fundamental rethinking of Integrated Fire Management (IFM). Recent frameworks, such as Law No. 15.228/2025 (Pantanal Law) and Law No. 14.944/2024 (National Policy for IFM), represent an important advance over traditional fire suppression. However, current IFM policies remain largely focused on managing fire within the boundaries of the biome (Oliveira et al. 2025). The evidence presented here indicates that a new generation of IFM should pursue two complementary objectives. First, it must mitigate the drivers of the transition itself by addressing the large‐scale processes that regulate the Pantanal's hydroclimatic functioning. This requires coordinated actions extending far beyond the wetland, including the conservation and restoration of native vegetation in the Upper Paraguay River Basin, the regulation of water use in the Planalto (Barros‐Rosa et al. 2025; Nunes et al. 2025), and the protection of Amazonian forests that sustain atmospheric moisture transport through climatic teleconnections (Bergier et al. 2018; Geirinhas et al. 2023). Maintaining hydrological and landscape connectivity across these coupled systems becomes as important as managing fuels locally (Tomas et al. 2019). In this perspective, the Pantanal serves as a crisis laboratory for tropical wetlands facing compounding hydroclimatic and anthropogenic pressures.

Second, IFM must recognize that some degree of hydroclimatic change is already unavoidable, requiring adaptation strategies capable of increasing the resilience of ecosystems and societies under an emerging fire regime (Rosenfield et al. 2025). This calls for transformative resilience within IFM, requiring substantial and explicit changes to social‐ecological systems. This integrates adaptive management, fast co‐learning, continuous monitoring, and early warning systems supported by AI and hydroclimatic indicators (such as BUI, VPD, and flood‐pulse duration). It also demands sub‐regionally tailored fire calendars (Garcia et al. 2021; Damasceno‐Junior et al. 2025), large‐scale ecological restoration, community‐based fire brigades, and flexible institutional arrangements capable of learning and responding to unprecedented environmental conditions (Pereira et al. 2025).

In synthesis, the fire regime transition can be seen as a challenge similar to other sustainability transitions, characterized by a process of change from one system state to another via a period of non‐linear disruptive change (Loorbach et al. 2017). In this context, IFM can no longer be conceived as a strategy for maintaining historical fire regimes alone. Instead, it should function as a dynamic governance system that continuously detects emerging transitions, anticipates ecological tipping points, and coordinates actions across scales to both reduce the likelihood of undesirable regime shifts and build societal capacity to navigate unavoidable transformations.

## Author Contributions

**Walfrido Moraes Tomas:** writing – review and editing, writing – original draft. **Pierre Girard:** conceptualization, writing – original draft, writing – review and editing. **Lucas Barros‐Rosa:** conceptualization, methodology, investigation, data curation, formal analysis, project administration, visualization, writing – review and editing, writing – original draft. **Renata Libonati:** methodology, validation, writing – review and editing, writing – original draft. **Priscila Lemes de Azevedo Silva:** conceptualization, writing – original draft, writing – review and editing. **Higo J. Dalmagro:** writing – review and editing. **Christine Strüssmann:** conceptualization, writing – original draft, writing – review and editing. **Thadeu Sobral de Souza:** methodology, validation, writing – review and editing, visualization, writing – original draft. **Mark Johnson:** conceptualization, writing – original draft, writing – review and editing. **Junior Gonçalves da Silva:** writing – review and editing. **Daniela de Oliveira Maionchi:** writing – review and editing, validation, methodology. **Liana O. Anderson:** writing – review and editing, writing – original draft. **Geraldo Alves Damasceno‐Júnior:** writing – review and editing, writing – original draft. **Fabio de Oliveira Roque:** conceptualization, writing – original draft, writing – review and editing, investigation. **Lúcia Mateus:** writing – review and editing, writing – original draft. **Jerry Penha:** conceptualization, investigation, writing – original draft, writing – review and editing, supervision, project administration. **Fernando A. da Costa Fernandes:** methodology, validation, writing – review and editing. **Cátia Nunes da Cunha:** writing – review and editing, writing – original draft.

## Conflicts of Interest

The authors declare no conflicts of interest.

## Supporting information


**Figure S1:** Trends and change points for Burned Area (BA) in the Northern Pantanal (1985–2024). Panels (a) and (b) show the observed values (orange bars) of BA on annual and monthly scales, respectively. The vertical dashed lines mark the detected significant change points. The solid black line represents the trend, with the gray area indicating the 95% confidence interval. The slope of significant trends (*p* < 0.05) is identified by ‘*’. The annual BA trend was not significant and was therefore modeled by LOESS, to demonstrate the potential non‐linear fluctuations in the series.
**Figure S2:** Trends and change points for Burned Area (BA) in the Central Pantanal (1985–2024). Panels (a) and (b) show the observed values (orange bars) of BA on annual and monthly scales, respectively. The vertical dashed lines mark the detected significant change points. The solid black line represents the trend, with the gray area indicating the 95% confidence interval. The slope of significant trends (*p* < 0.05) is identified by ‘*’. The annual BA trend was not significant and was therefore modeled by LOESS, to demonstrate the potential non‐linear fluctuations in the series.
**Figure S3:** Trends and change points for Burned Area (BA) in the Southern Pantanal (1985–2024). Panels (a) and (b) show the observed values (orange bars) of BA on annual and monthly scales, respectively. The vertical dashed lines mark the detected significant change points. The solid black line represents the trend, with the gray area indicating the 95% confidence interval. The trends in the annual and monthly BA time series were not significant (*p* < 0.05), and were therefore modeled by LOESS, to demonstrate the potential non‐linear fluctuations in the series.
**Figure S4:** Relationship of Vapor Pressure Deficit (VPD) with Burned Area (BA), VPD trend and its correlation with the Buildup Index (BUI). (a) and (d) show relationships between VPD and the fire variables monthly BA and annual MFS, respectively. The red dots indicate BA months classified as extreme (95th percentile). (b) and (e) show long‐term trends of VPD (monthly and annual), analyzed by the Modified Mann‐Kendall test. (c) and (f) show the correlation between VPD and the Buildup Index BUI on monthly and annual scales.
**Table S1:** Summary of the three Pantanal sectors used in the sub‐regional analyses. The grouping follows the physiographic sub‐regions of Silva and Abdon (1998) and reflects the main north–south hydrological gradient of the flood pulse. Provides the rationale for grouping the 11 physiographic sub‐regions of the Pantanal into three broader analytical sectors: Northern, Central, and Southern Pantanal. This grouping was based on the main north–south hydrological gradient of the flood pulse, from earlier inundation in the northern floodplain to delayed flooding in the southern sector, as well as broad differences in geomorphology, vegetation, soil, floodplain structure, and fuel conditions. This subdivision allowed us to compare fire dynamics under contrasting hydrological contexts while avoiding excessive fragmentation of the time‐series analyses into smaller physiographic units.
**Table S2:** Results of the simple regression models between monthly burned area individual predictors. ‘*n*’ indicates the number of monthly observations used in each regression after accounting for predictor‐specific lags. The table lists the results of the Simple Regression analysis for all predictors tested in relation to monthly BA (log10 ha), ordered in descending order by *R* ^2^. *R* ^2^ quantifies the individual predictive power of each variable. The ‘Model’ column indicates the best‐fit function used (Linear or Exponential Saturation), and Slope provides the magnitude and direction of the relationship between the predictor and BA.
**Table S3:** Relative importance of all predictors in the Elastic Net Model for monthly burned area. The importance of each variable was estimated from the absolute values of the standardized regression coefficients of the Elastic Net model. This value reflects the strength of each predictor's contribution to the variance of the response variable.
**Table S4:** Cross‐Validation and temporal partitioning metrics for the monthly burned area Elastic Net model in the Northern Pantanal. The table details the training and test partitions used in each of the five folds for the evaluation of the model's temporal stability.
**Table S5:** Cross‐Validation and temporal partitioning metrics for the monthly burned area Elastic Net model in the Central Pantanal. The table details the training and test partitions used in each of the five folds for the evaluation of the model's temporal stability.
**Table S6:** Cross‐Validation and temporal partitioning metrics for the monthly burned area Elastic Net model in the Southern Pantanal. The table details the training and test partitions used in each of the five folds for the evaluation of the model's temporal stability.
**Table S7:** Relative importance of all predictors in the Elastic Net Model for monthly burned area in the Northern Pantanal. The importance of each variable was estimated from the absolute values of the standardized regression coefficients of the Elastic Net model. This value reflects the strength of each predictor's contribution to the variance of the response variable.
**Table S8:** Relative importance of all predictors in the Elastic Net Model for monthly burned area in the Central Pantanal. The importance of each variable was estimated from the absolute values of the standardized regression coefficients of the Elastic Net model. This value reflects the strength of each predictor's contribution to the variance of the response variable.
**Table S9:** Relative importance of all predictors in the Elastic Net Model for monthly burned area in the Southern Pantanal. The importance of each variable was estimated from the absolute values of the standardized regression coefficients of the Elastic Net model. This value reflects the strength of each predictor's contribution to the variance of the response variable.

## Data Availability

The data that support the findings of this study are available in Zenodo at https://doi.org/10.5281/zenodo.20060087. These data were derived from the following resources available in the public domain—MapBiomas Fire, https://doi.org/10.58053/MapBiomas/8JLX1T MapBiomas LULC, https://doi.org/10.58053/MapBiomas/XXUKA8 ECMWF (ERA5), https://doi.org/10.24381/cds.f17050d7 ECMWF (CEMS), https://doi.org/10.24381/cds.0e89c522.

## References

[gcb71089-bib-0001] Ab'Saber, A. N. 1988. “O Pantanal Mato‐Grossense e a Teoria Dos Refúgios.” Revista Brasileira de Geografia 50, no. 2: 9–57.

[gcb71089-bib-0002] Alencar, A. A. , V. L. Arruda , W. V. D. Silva , et al. 2022. “Long‐Term Landsat‐Based Monthly Burned Area Dataset for the Brazilian Biomes Using Deep Learning.” Remote Sensing 14, no. 11: 2510. 10.3390/rs14112510.

[gcb71089-bib-0003] Aoki, C. , R. R. Faria , G. A. Damasceno‐Junior , and A. Pott . 2021. “Synthesis of the Present Knowledge on Plant Phenology of the Pantanal.” In Flora and Vegetation of the Pantanal Wetland, edited by G. A. Damasceno‐Junior and A. Pott , vol. 18, 535–549. Springer International Publishing. 10.1007/978-3-030-83375-6_13.

[gcb71089-bib-0004] Archibald, S. , C. E. Lehmann , J. L. Gómez‐Dans , and R. A. Bradstock . 2013. “Defining Pyromes and Global Syndromes of Fire Regimes.” Proceedings of the National Academy of Sciences of the United States of America 110, no. 16: 6442–6447. 10.1073/pnas.1211466110. 23559374 PMC3631631

[gcb71089-bib-0005] Archibald, S. , C. E. R. Lehmann , C. M. Belcher , et al. 2018. “Biological and Geophysical Feedbacks With Fire in the Earth System.” Environmental Research Letters 13, no. 3: 033003. 10.1088/1748-9326/aa9ead.

[gcb71089-bib-0006] Arruda, W. D. S. , J. Oldeland , A. C. Paranhos Filho , et al. 2016. “Inundation and Fire Shape the Structure of Riparian Forests in the Pantanal, Brazil.” PLoS One 11, no. 6: e0156825. 10.1371/journal.pone.0156825. 27280879 PMC4900580

[gcb71089-bib-0007] Assine, M. L. , H. A. Macedo , J. C. Stevaux , I. Bergier , C. R. Padovani , and A. Silva . 2015. “Avulsive Rivers in the Hydrology of the Pantanal Wetland.” In Dynamics of the Pantanal Wetland in South America, 83–110. Springer. 10.1007/698_2015_351.

[gcb71089-bib-0008] Balch, J. K. , P. M. Brando , D. C. Nepstad , et al. 2015. “The Susceptibility of Southeastern Amazon Forests to Fire: Insights From a Large‐Scale Burn Experiment.” Bioscience 65, no. 9: 893–905. 10.1093/biosci/biv106.

[gcb71089-bib-0009] Barros‐Rosa, L. , P. H. Z. de Arruda , N. G. Machado , J. C. Pires‐Oliveira , and P. V. Eisenlohr . 2022. “Fire Probability Mapping and Prediction From Environmental Data: What a Comprehensive Savanna‐Forest Transition Can Tell Us.” Forest Ecology and Management 520: 120354. 10.1016/j.foreco.2022.120354.

[gcb71089-bib-0010] Barros‐Rosa, L. , L. M. Peluso , P. Lemes , et al. 2025. “The Ineffectiveness of Current Environmental and Fire Policies in the World's Largest Wetland.” Environmental Research Letters 20, no. 3: 034039.

[gcb71089-bib-0011] Belém, L. B. C. , R. Albuquerque , L. F. Peres , et al. 2026. “Dry–Hot Compound Events Driving the 2024 Pantanal Wildfires.” International Journal of Climatology 46: e70312. 10.1002/joc.70312.

[gcb71089-bib-0012] Bergier, I. 2013. “Effects of Highland Land‐Use Over Lowlands of the Brazilian Pantanal.” Science of the Total Environment 463: 1060–1066. 10.1016/j.scitotenv.2013.06.036. 23891998

[gcb71089-bib-0013] Bergier, I. , M. L. Assine , M. M. McGlue , et al. 2018. “Amazon Rainforest Modulation of Water Security in the Pantanal Wetland.” Science of the Total Environment 619: 1116–1125. 10.1016/j.scitotenv.2017.11.163. 29734590

[gcb71089-bib-0014] Bergstra, J. , and Y. Bengio . 2012. “Random Search for Hyper‐Parameter Optimization.” Journal of Machine Learning Research 13, no. 2: 281–305. 10.1088/1748-9326/adb7f5.

[gcb71089-bib-0015] Botelho, M. T. D. A. , R. Morais Chiaravalloti , and C. Niel Berlinck . 2025. “Playing With Fire: The Vital Influence of Traditional Knowledge on the Socio Biodiversity of the Pantanal.” Biodiversidade Brasileira 14, no. 4: 155–168. 10.37002/biodiversidadebrasileira.v14i4.2547.

[gcb71089-bib-0016] Box, G. E. , and D. R. Cox . 1964. “An Analysis of Transformations.” Journal of the Royal Statistical Society. Series B, Statistical Methodology 26, no. 2: 211–243.

[gcb71089-bib-0017] Brazil . 2024. “Law No. 14,944 of July 31, 2024. Establishes the National Policy for Integrated Fire Management. Official Gazette of the Federative Republic of Brazil.”

[gcb71089-bib-0018] Brazil . 2025. “Law No. 15,228 of September 30, 2025. Provides for the Use, Conservation, Protection, and Recovery of the Pantanal Biome. Official Gazette of the Federative Republic of Brazil.”

[gcb71089-bib-0019] Caballero, C. B. , T. W. Biggs , N. Vergopolan , et al. 2025. “Decadal Hydroclimatic Changes in the Pantanal, the World's Largest Tropical Wetland.” Scientific Reports 15, no. 1: 17675. 10.1038/s41598-025-01980-6. 40399366 PMC12095462

[gcb71089-bib-0020] Cawson, J. G. , L. Collins , S. A. Parks , R. H. Nolan , and T. D. Penman . 2024. “Atmospheric Dryness Removes Barriers to the Development of Large Forest Fires.” Agricultural and Forest Meteorology 350: 109990. 10.1016/j.agrformet.2024.109990.

[gcb71089-bib-0021] Chatterjee, S. , and A. S. Hadi . 2015. Regression Analysis by Example. John Wiley & Sons.

[gcb71089-bib-0022] Cleveland, W. S. 1979. “Robust Locally Weighted Regression and Smoothing Scatterplots.” Journal of the American Statistical Association 74, no. 368: 829–836.

[gcb71089-bib-0023] Convention on Wetlands . 2025. Global Wetland Outlook 2025: Valuing, Conserving, Restoring and Financing Wetlands. Secretariat of the Convention on Wetlands. 10.69556/GWO-2025-eng.

[gcb71089-bib-0024] Correa, D. B. , M. G. Fonseca , R. Libonati , et al. 2022. “Increased Burned Area in the Pantanal Over the Past Two Decades.” Science of the Total Environment 835: 155386. 10.1016/j.scitotenv.2022.155386. 35461933

[gcb71089-bib-0026] Damasceno‐Junior, G. A. , A. D. M. M. Pereira , J. Oldeland , P. Parolin , and A. Pott . 2021. “Fire, Flood and Pantanal Vegetation.” In Flora and Vegetation of the Pantanal Wetland, edited by G. A. Damasceno‐Junior and A. Pott , vol. 18, 661–688. Springer International Publishing. 10.1007/978-3-030-83375-6_18.

[gcb71089-bib-0027] Damasceno‐Junior, G. A. , A. D. M. M. Pereira , B. H. Saharjo , et al. 2025. “Flood‐Fire Interplays in Wetlands: The Rising of an Actionable Field of Study.” Wetlands 45, no. 8: 122. 10.1007/s13157-025-02005-8.

[gcb71089-bib-0028] Dormann, C. F. , J. Elith , S. Bacher , et al. 2013. “Collinearity: A Review of Methods to Deal With It and a Simulation Study Evaluating Their Performance.” Ecography 36, no. 1: 27–46. 10.1111/j.1600-0587.2012.07348.x.

[gcb71089-bib-0029] Dunn, P. K. , and G. K. Smyth . 2005. “Series Evaluation of Tweedie Exponential Dispersion Model Densities.” Statistics and Computing 15, no. 4: 267–280. 10.1007/s11222-005-4070-y.

[gcb71089-bib-0030] Fann, N. , B. Alman , R. A. Broome , et al. 2018. “The Health Impacts and Economic Value of Wildland Fire Episodes in the US: 2008–2012.” Science of the Total Environment 610: 802–809. 10.1016/j.scitotenv.2017.08.024. 28826118 PMC6117838

[gcb71089-bib-0031] Faria, B. L. , A. Staal , C. A. Silva , P. A. Martin , P. K. Panday , and V. L. Dantas . 2021. “Climate Change and Deforestation Increase the Vulnerability of Amazonian Forests to Post‐Fire Grass Invasion.” Global Ecology and Biogeography 30, no. 12: 2368–2381. 10.1111/geb.13388.

[gcb71089-bib-0032] Garcia, L. C. , J. K. Szabo , F. de Oliveira Roque , et al. 2021. “Record‐Breaking Wildfires in the World's Largest Continuous Tropical Wetland: Integrative Fire Management is Urgently Needed for Both Biodiversity and Humans.” Journal of Environmental Management 293: 112870. 10.1016/j.jenvman.2021.112870. 34052615

[gcb71089-bib-0033] García, M. , M. L. Pettinari , E. Chuvieco , et al. 2022. “Characterizing Global Fire Regimes From Satellite‐Derived Products.” Forests 13, no. 5: 699. 10.3390/f13050699.

[gcb71089-bib-0034] Geirinhas, J. L. , A. C. Russo , R. Libonati , et al. 2023. “Combined Large‐Scale Tropical and Subtropical Forcing on the Severe 2019–2022 Drought in South America.” npj Climate and Atmospheric Science 6, no. 1: 185. 10.1038/s41612-023-00510-3.

[gcb71089-bib-0035] Hair, J. F. , W. C. Black , B. J. Babin , and R. E. Anderson . 2013. Multivariate Data Analysis. Pearson Higher Ed.

[gcb71089-bib-0036] Hamed, K. H. , and A. R. Rao . 1998. “A Modified Mann‐Kendall Trend Test for Autocorrelated Data.” Journal of Hydrology 204: 182–196. 10.1016/S0022-1694(97)00125-X.

[gcb71089-bib-0101] Hamilton, S. K. , S. J. Sippel , and J. M. Melack . 1996. “Inundation Patterns in the Pantanal Wetland of South America Determined From Passive Microwave Remote Sensing.” Archiv für Hydrobiologie 137, no. 1: 1–23. 10.1127/archiv-hydrobiol/137/1996/1.

[gcb71089-bib-0037] He, H. , and Y. Ma . 2013. Imbalanced Learning: Foundations, Algorithms, and Applications. Wiley Ed. Wiley.

[gcb71089-bib-0038] Hersbach, H. , B. Bell , P. Berrisford , et al. 2020. “The ERA5 Global Reanalysis.” Quarterly Journal of the Royal Meteorological Society 146, no. 730: 1999–2049. 10.1002/qj.3803.

[gcb71089-bib-0039] Hurteau, M. D. , J. B. Bradford , P. Z. Fulé , A. H. Taylor , and K. L. Martin . 2014. “Climate Change, Fire Management, and Ecological Services in the Southwestern US.” Forest Ecology and Management 327: 280–289. 10.1016/j.foreco.2013.08.007.

[gcb71089-bib-0040] IBGE‐Instituto Brasileiro de Geografia e Estatística . 2025. “Biomas e Sistema Costeiro‐Marinho do Brasil (Escala 1:250.000). Portal de Geociências.” https://www.ibge.gov.br/geociencias/cartas‐e‐mapas/informacoes‐ambientais/15842‐biomas.html.

[gcb71089-bib-0041] Jain, P. , S. C. Coogan , S. G. Subramanian , M. Crowley , S. Taylor , and M. D. Flannigan . 2020. “A Review of Machine Learning Applications in Wildfire Science and Management.” Environmental Reviews 28, no. 4: 478–505. 10.1139/er-2020-0019.

[gcb71089-bib-0043] Junk, W. J. , and C. N. Cunha . 2012. “Pasture Clearing From Invasive Woody Plants in the Pantanal: A Tool for Sustainable Management or Environmental Destruction?” Wetlands Ecology and Management 20, no. 2: 111–122. 10.1007/s11273-011-9246-y.

[gcb71089-bib-0044] Junk, W. J. , and C. Nunes da Cunha . 2016. “The Pantanal: A Brief Review of Its Ecology, Biodiversity, and Protection Status.” In The Wetland Book, edited by C. Finlayson , G. Milton , R. Prentice , and N. Davidson . Springer. 10.1007/978-94-007-6173-5_129-1.

[gcb71089-bib-0045] Kelly, L. T. , K. M. Giljohann , A. Duane , et al. 2020. “Fire and Biodiversity in the Anthropocene.” Science 370, no. 6519: eabb0355. 10.1126/science.abb0355. 33214246

[gcb71089-bib-0046] Khodaee, M. , K. Easterday , and K. Klausmeyer . 2024. “Integrating Hydrological Parameters in Wildfire Risk Assessment: A Machine Learning Approach for Mapping Wildfire Probability.” Environmental Research Letters 19, no. 11: 114043. 10.1088/1748-9326/ad80ad.

[gcb71089-bib-0047] Kottek, M. , J. Grieser , C. Beck , B. Rudolf , and F. Rubel . 2006. “World Map of the Köppen‐Geiger Climate Classification Updated.” Meteorologische Zeitschrift 15, no. 3: 259–263. 10.1127/0941-2948/2006/0130.

[gcb71089-bib-0048] Kotze, D. C. 2013. “The Effects of Fire on Wetland Structure and Functioning.” African Journal of Aquatic Science 38, no. 3: 237–247.

[gcb71089-bib-0049] Krebs, P. , G. B. Pezzatti , S. Mazzoleni , L. M. Talbot , and M. Conedera . 2010. “Fire Regime: History and Definition of a Key Concept in Disturbance Ecology.” Theory in Biosciences 129, no. 1: 53–69. 10.1007/s12064-010-0082-z. 20502984

[gcb71089-bib-0050] Kuhn, M. , and K. Johnson . 2013. Applied Predictive Modeling. Vol. 26, 13. Springer.

[gcb71089-bib-0051] Kumar, S. , A. Getirana , R. Libonati , C. Hain , S. Mahanama , and N. Andela . 2022. “Changes in Land Use Enhance the Sensitivity of Tropical Ecosystems to Fire‐Climate Extremes.” Scientific Reports 12: 964. 10.1038/s41598-022-05130-0. 35046481 PMC8770517

[gcb71089-bib-0052] Leeds, J. A. , P. B. Garrett , and J. M. Newman . 2009. “Assessing Impacts of Hydropattern Restoration of an Overdrained Wetland on Soil Nutrients, Vegetation and Fire.” Restoration Ecology 17, no. 4: 460–469. 10.1111/j.1526-100X.2008.00381.x.

[gcb71089-bib-0053] Libonati, R. , C. C. DaCamara , L. F. Peres , L. A. Sander De Carvalho , and L. C. Garcia . 2020. “Rescue Brazil's Burning Pantanal Wetlands.” Nature 588, no. 7837: 217–219. 10.1038/d41586-020-03464-1. 33293715

[gcb71089-bib-0054] Libonati, R. , J. L. Geirinhas , P. S. Silva , et al. 2022. “Assessing the Role of Compound Drought and Heatwave Events on Unprecedented 2020 Wildfires in the Pantanal.” Environmental Research Letters 17, no. 1: 015005. 10.1088/1748-9326/ac462e.

[gcb71089-bib-0055] Loorbach, D. , N. Frantzeskaki , and F. Avelino . 2017. “Sustainability Transitions Research: Transforming Science and Practice for Societal Change.” Annual Review of Environment and Resources 42: 599–626. 10.1146/annurev-environ-102014-021340.

[gcb71089-bib-0056] Lorenz, C. , R. Libonati , L. B. C. Belém , et al. 2024. “Historical Association Between Respiratory Diseases Hospitalizations and Fire Occurrence in the Pantanal Wetland, Brazil.” Atmospheric Pollution Research 15, no. 8: 102182. 10.1016/j.apr.2024.102182.

[gcb71089-bib-0057] Luković, J. , J. C. Chiang , D. Blagojević , and A. Sekulić . 2021. “A Later Onset of the Rainy Season in California.” Geophysical Research Letters 48, no. 4: e2020GL090350. 10.1029/2020GL090350.

[gcb71089-bib-0058] MacFarland, T. W. , and J. M. Yates . 2016. “Mann–Whitney u Test.” In Introduction to Nonparametric Statistics for the Biological Sciences Using R, 103–132. Springer International Publishing. 10.1007/978-3-319-30634-6_4.

[gcb71089-bib-0059] Marengo, J. A. , L. M. Alves , and R. R. Torres . 2016. “Regional Climate Change Scenarios in the Brazilian Pantanal Watershed.” Climate Research 68, no. 2–3: 201–213. 10.3354/cr01324.

[gcb71089-bib-0060] Marengo, J. A. , A. P. Cunha , L. A. Cuartas , et al. 2021. “Extreme Drought in the Brazilian Pantanal in 2019–2020: Characterization, Causes, and Impacts.” Frontiers in Water 3: 13. 10.3389/frwa.2021.639204.

[gcb71089-bib-0061] Marengo, J. A. , G. S. Oliveira , and L. M. Alves . 2015. “Climate Change Scenarios in the Pantanal.” In Dynamics of the Pantanal Wetland in South America, 227–238. Springer. 10.1007/698_2015_357.

[gcb71089-bib-0062] Marques, J. F. , M. B. Alves , C. F. Silveira , et al. 2021. “Fires Dynamics in the Pantanal: Impacts of Anthropogenic Activities and Climate Change.” Journal of Environmental Management 299: 113586. 10.1016/j.jenvman.2021.113586. 34454200

[gcb71089-bib-0063] McKee, T. B. , N. J. Doesken , and J. Kleist . 1993. “The Relationship of Drought Frequency and Duration to Time Scales.” Proceedings of the 8th Conference on Applied Climatology 17, no. 22: 179–183.

[gcb71089-bib-0064] McWethy, D. B. , T. Schoennagel , P. E. Higuera , et al. 2019. “Rethinking Resilience to Wildfire.” Nature Sustainability 2, no. 9: 797–804. 10.1038/s41893-019-0353-8.

[gcb71089-bib-0065] Menezes, L. S. , A. M. de Oliveira , F. L. Santos , et al. 2022. “Lightning Patterns in the Pantanal: Untangling Natural and Anthropogenic‐Induced Wildfires.” Science of the Total Environment 820: 153021. 10.1016/j.scitotenv.2022.153021. 35026277

[gcb71089-bib-0066] Muggeo, V. M. 2003. “Estimating Regression Models With Unknown Break‐Points.” Statistics in Medicine 22, no. 19: 3055–3071. 10.1002/sim.1545. 12973787

[gcb71089-bib-0067] Nunes, N. C. , N. G. Machado , L. Barros‐Rosa , L. O. F. Dos Santos , F. A. C. Fernandes , and M. S. Biudes . 2025. “Land Use Change and Water Loss in the Upper Paraguay River Basin: Trends, Future Scenarios, and Implications for the Pantanal.” Journal of Environmental Management 394: 127666. 10.1016/j.jenvman.2025.127666. 41100943

[gcb71089-bib-0068] Oliveira, M. D. R. , A. D. M. M. Pereira , F. Bao , et al. 2025. “Designing Burn Windows for Integrated Fire Management in Wetlands: Why Should Flooding Not Be Overlooked?” Wetlands 45, no. 4: 35. 10.1007/s13157-025-01919-7.

[gcb71089-bib-0069] Oliveira Vieira, S. , P. Satyamurty , and R. V. Andreoli . 2013. “On the South Atlantic Convergence Zone Affecting Southern Amazonia in Austral Summer.” Atmospheric Science Letters 14, no. 1: 1–6. 10.1002/asl2.401.

[gcb71089-bib-0070] Page, S. E. , F. Siegert , J. O. Rieley , H. D. V. Boehm , A. Jaya , and S. Limin . 2002. “The Amount of Carbon Released From Peat and Forest Fires in Indonesia During 1997.” Nature 420, no. 6911: 61–65. 10.1038/nature01131. 12422213

[gcb71089-bib-0071] Pausas, J. G. , and J. E. Keeley . 2014. “Abrupt Climate‐Independent Fire Regime Changes.” Ecosystems 17, no. 6: 1109–1120. 10.1007/s10021-014-9773-5.

[gcb71089-bib-0072] Pausas, J. G. , and J. E. Keeley . 2019. “Wildfires as an Ecosystem Service.” Frontiers in Ecology and the Environment 17, no. 5: 289–295. 10.1002/fee.2044.

[gcb71089-bib-0073] Pereira, A. D. M. M. , G. A. Damasceno‐Junior , R. Libonati , et al. 2025. “Catastrophic Wildfires in the Pantanal Wetlands as Catalysts for Transformative Change.” Environmental Science & Policy 174: 104273. 10.1016/j.envsci.2025.104273.

[gcb71089-bib-0074] Pereira, G. , R. D. C. Ramos , L. C. Rocha , et al. 2021. “Rainfall Patterns and Geomorphological Controls Driving Inundation Frequency in Tropical Wetlands: How Does the Pantanal Flood?” Progress in Physical Geography: Earth and Environment 45, no. 5: 669–686. 10.1177/0309133320987719.

[gcb71089-bib-0075] Pivello, V. R. , I. Vieira , A. V. Christianini , et al. 2021. “Understanding Brazil's Catastrophic Fires: Causes, Consequences and Policy Needed to Prevent Future Tragedies.” Perspectives in Ecology and Conservation 19: 233–255. 10.1016/j.pecon.2021.06.005.

[gcb71089-bib-0076] Pott, A. , and J. S. V. da Silva . 2015. “Terrestrial and Aquatic Vegetation Diversity of the Pantanal Wetland.” In Dynamics of the Pantanal Wetland in South America. The Handbook of Environmental Chemistry, edited by I. Bergier and M. Assine , vol. 37. Springer. 10.1007/698_2015_352.

[gcb71089-bib-0077] Rosenfield, M. F. , L. Jardim , M. Antongiovanni , et al. 2025. “Mapping Resilient Landscapes to Climate Change in a Megadiverse Country.” Global Change Biology 31, no. 10: e70544. 10.1111/gcb.70544. 41070834 PMC12512505

[gcb71089-bib-0078] Scheffer, M. , S. Carpenter , J. A. Foley , C. Folke , and B. Walker . 2001. “Catastrophic Shifts in Ecosystems.” Nature 413, no. 6856: 591–596. 10.1038/35098000. 11595939

[gcb71089-bib-0079] Scheffer, M. , and S. R. Carpenter . 2003. “Catastrophic Regime Shifts in Ecosystems: Linking Theory to Observation.” Trends in Ecology & Evolution 18, no. 12: 648–656. 10.1016/j.tree.2003.09.002.

[gcb71089-bib-0080] Sen, P. K. 1968. “Estimates of the Regression Coefficient Based on Kendall's Tau.” Journal of the American Statistical Association 63: 1379–1389.

[gcb71089-bib-0081] Shojaei, A. , and I. Flood . 2018. “Univariate Modeling of the Timings and Costs of Unknown Future Project Streams: A Case Study.” International Journal on Advances in Systems and Measurements 11: 36–46.

[gcb71089-bib-0082] Silva, J. D. S. V. , and M. M. Abdon . 1998. “Delimitação do Pantanal Brasileiro e Suas Sub‐Regiões.” Pesquisa Agropecuária Brasileira 33: 1703–1711.

[gcb71089-bib-0083] Silva, M. P. D. , R. Mauro , G. Mourao , and M. Coutinho . 2000. “Distribuição e Quantificação de Classes de Vegetação do Pantanal Através de Levantamento Aéreo.” Revista Brasileira de Botânica 23, no. 2: 143–152. 10.1590/S0100-84042000000200004.

[gcb71089-bib-0084] Silva, P. S. , J. L. Geirinhas , R. Lapere , et al. 2022. “Heatwaves and Fire in Pantanal: Historical and Future Perspectives From CORDEX‐CORE.” Journal of Environmental Management 323: 116193. 10.1016/j.jenvman.2022.116193. 36150352

[gcb71089-bib-0085] Souza, C. M. Z., Jr. , J. Shimbo , M. R. Rosa , et al. 2020. “Reconstructing Three Decades of Land Use and Land Cover Changes in Brazilian Biomes With Landsat Archive and Earth Engine.” Remote Sensing 12, no. 17: 2735. 10.3390/rs12172735.

[gcb71089-bib-0086] Spinoni, J. , G. Naumann , H. Carrao , P. Barbosa , and J. Vogt . 2014. “World Drought Frequency, Duration, and Severity for 1951–2010.” International Journal of Climatology 34, no. 8: 2792–2804. 10.1002/joc.3875.

[gcb71089-bib-0087] Spinoni, J. , G. Naumann , J. Vogt , and P. Barbosa . 2015. “European Drought Climatologies and Trends Based on a Multi‐Indicator Approach.” Global and Planetary Change 127: 50–57. 10.1016/j.gloplacha.2015.01.012.

[gcb71089-bib-0088] Stevaux, J. C. , H. de Azevedo Macedo , M. L. Assine , and A. Silva . 2020. “Changing Fluvial Styles and Backwater Flooding Along the Upper Paraguay River Plains in the Brazilian Pantanal Wetland.” Geomorphology 350: 106906. 10.1016/j.geomorph.2019.106906.

[gcb71089-bib-0089] Tetens, O. 1930. “Uber Einige Meteorologische Begriffe.” Zeitschrift für Geophysik 6: 297–309.

[gcb71089-bib-0090] Thielen, D. , K. L. Schuchmann , P. Ramoni‐Perazzi , et al. 2020. “Quo Vadis Pantanal? Expected Precipitation Extremes and Drought Dynamics From Changing Sea Surface Temperature.” PLoS One 15, no. 1: e0227437. 10.1371/journal.pone.0227437. 31910441 PMC6946591

[gcb71089-bib-0091] Tomas, W. M. , C. N. Berlinck , R. M. Chiaravalloti , et al. 2021. “Distance Sampling Surveys Reveal 17 Million Vertebrates Directly Killed by the 2020's Wildfires in the Pantanal, Brazil.” Scientific Reports 11, no. 1: 23547. 10.1038/s41598-021-02844-5. 34916541 PMC8677733

[gcb71089-bib-0092] Tomas, W. M. , F. de Oliveira Roque , R. G. Morato , et al. 2019. “Sustainability Agenda for the Pantanal Wetland: Perspectives on a Collaborative Interface for Science, Policy, and Decision‐Making.” Tropical Conservation Science 12: 1940082919872634. 10.1177/1940082919872634.

[gcb71089-bib-0093] Tortato, F. R. , T. J. Izzo , R. Hoogesteijn , and C. A. Peres . 2017. “The Numbers of the Beast: Valuation of Jaguar ( *Panthera onca* ) Tourism and Cattle Depredation in the Brazilian Pantanal.” Global Ecology and Conservation 11: 106–114. 10.1016/j.gecco.2017.05.003.

[gcb71089-bib-0094] Van der Werf, G. R. , J. T. Randerson , L. Giglio , et al. 2017. “Global Fire Emissions Estimates During 1997–2016.” Earth System Science Data 9, no. 2: 697–720. 10.5194/essd-9-697-2017.

[gcb71089-bib-0095] Vasconcelos, R. N. , M. M. M. de Santana , D. P. Costa , et al. 2025. “Machine Learning Model Reveals Land Use and Climate's Role in Caatinga Wildfires: Present and Future Scenarios.” Fire 8, no. 1: 8. 10.3390/fire8010008.

[gcb71089-bib-0096] Veiga, R. M. , C. von Randow , C. Burton , D. I. Kelley , M. Cardoso , and F. Morelli . 2025. “Fire Emissions in the Brazilian Cerrado–Dynamics, Estimates, Management, and Their Role in the Global Carbon Budget.” Natural Hazards and Earth System Sciences 25, no. 9: 3581–3601.

[gcb71089-bib-0097] Vitolo, C. , F. Di Giuseppe , C. Barnard , et al. 2020. “ERA5‐Based Global Meteorological Wildfire Danger Maps.” Scientific Data 7: 216. 10.1038/s41597-020-0554-z. 32636392 PMC7341852

[gcb71089-bib-0098] Yuan, H. , F. Restuccia , and G. Rein . 2021. “Spontaneous Ignition of Soils: A Multi‐Step Reaction Scheme to Simulate Self‐Heating Ignition of Smouldering Peat Fires.” International Journal of Wildland Fire 30, no. 6: 440–453. 10.1071/WF19128.

[gcb71089-bib-0100] Zou, H. , and T. Hastie . 2005. “Regularization and Variable Selection via the Elastic Net.” Journal of the Royal Statistical Society, Series B: Statistical Methodology 67, no. 2: 301–320. 10.1111/j.1467-9868.2005.00503.x.

