# Supplementary material for: Climatic and Hydrological Changes Drive Contemporary Fire Regime Transitions in the World's Largest Wetland
Source: Glob Chang Biol. 2026 Sep 4;32(9):e71089. doi: 10.1111/gcb.71089 (PMC13545500; doi:10.1111/gcb.71089)
Supplement: Supplementary file 1 [file GCB-32-e71089-s001.docx]

**Supplementary Material**

 **Figure S1. Trends and change points for Burned Area (BA) in the Northern Pantanal (1985-2024).** Panels (A) and (B) show the observed values (orange bars) of BA on annual and monthly scales, respectively. The vertical dashed lines mark the detected significant change points. The solid black line represents the trend, with the gray area indicating the 95% confidence interval. The slope of significant trends (p < 0.05) is identified by ‘*’. The annual BA trend was not significant and was therefore modeled by LOESS, to demonstrate the potential non-linear fluctuations in the series.

**Figure S2. Trends and change points for Burned Area (BA) in the Central Pantanal (1985-2024).** Panels (A) and (B) show the observed values (orange bars) of BA on annual and monthly scales, respectively. The vertical dashed lines mark the detected significant change points. The solid black line represents the trend, with the gray area indicating the 95% confidence interval. The slope of significant trends (p < 0.05) is identified by ‘*’. The annual BA trend was not significant and was therefore modeled by LOESS, to demonstrate the potential non-linear fluctuations in the series.

**Figure S3. Trends and change points for Burned Area (BA) in the Southern Pantanal (1985-2024).** Panels (A) and (B) show the observed values (orange bars) of BA on annual and monthly scales, respectively. The vertical dashed lines mark the detected significant change points. The solid black line represents the trend, with the gray area indicating the 95% confidence interval. The trends in the annual and monthly BA time series were not significant (p < 0.05), and were therefore modeled by LOESS, to demonstrate the potential non-linear fluctuations in the series.

**Figure S4. Relationship of Vapor Pressure Deficit (VPD) with Burned Area (BA), VPD trend and its correlation with the Buildup Index (BUI). (**A) and (D) show relationships between VPD and the fire variables monthly BA and annual MFS, respectively. The red dots indicate BA months classified as extreme (95th percentile). (B) and (E) show long-term trends of VPD (monthly and annual), analyzed by the Modified Mann-Kendall test. (C) and (F) show the correlation between VPD and the Buildup Index BUI on monthly and annual scales.

**Table S1. Summary of the three Pantanal sectors used in the sub-regional analyses. The grouping follows the physiographic sub-regions of Silva and Abdon (1998) and reflects the main north-south hydrological gradient of the flood pulse.** Provides the rationale for grouping the 11 physiographic sub-regions of the Pantanal into three broader analytical sectors: Northern, Central, and Southern Pantanal. This grouping was based on the main north–south hydrological gradient of the flood pulse, from earlier inundation in the northern floodplain to delayed flooding in the southern sector, as well as broad differences in geomorphology, vegetation, soil, floodplain structure, and fuel conditions. This subdivision allowed us to compare fire dynamics under contrasting hydrological contexts while avoiding excessive fragmentation of the time-series analyses into smaller physiographic units.

| **Analytical sector** | **Grouped physiographic sub-regions** | **Main basis for grouping** | **Key hydroclimatic and environmental characteristics** | **Relevance for fire-regime analysis** | **Supporting references** |
| --- | --- | --- | --- | --- | --- |
| **Northern Pantanal** | Cáceres, Poconé, and Barão de Melgaço | Represents the upper/northern floodplain sector, closer to the main upstream inflow areas of the Upper Paraguay River Basin. | This sector is more directly influenced by earlier seasonal flooding, with maximum inundation occurring earlier than in the central and southern portions of the floodplain. It includes extensive floodplain areas where seasonal inundation strongly controls fuel exposure and the transition between wet and dry phases. | Fire activity in this sector is expected to be strongly constrained by the timing and duration of the flood pulse. Earlier flooding can reduce fuel exposure during part of the year, while delayed rainfall or shortened inundation may rapidly increase flammability. | Silva and Abdon (1998); Hamilton et al. (1996); Marengo et al. (2021); Stevaux et al. (2020) |
| **Central Pantanal** | Paraguai, Paiaguás, and Nhecolândia | Represents the broad central low-gradient floodplain, including large floodplain units and heterogeneous flooded/non-flooded mosaics. | This sector includes some of the largest Pantanal sub-regions and is characterized by broad plains, seasonal water accumulation, sandy soils in several areas, and a mosaic of grasslands, savannas, forest patches, and aquatic habitats. Vegetation distribution is strongly related to inundation depth, soil, and topographic position. | The central sector functions as a transitional hydrological and fuel-condition zone. Because it combines large open floodplain areas with heterogeneous vegetation mosaics, changes in flood duration, atmospheric dryness, and rainfall timing may strongly affect fire-season duration and fire spread. | Silva and Abdon (1998); Silva et al. (2000); Hamilton et al. (1996); Stevaux et al. (2020); Thielen et al. (2020) |
| **Southern Pantanal** | Nabileque, Porto Murtinho, Miranda, Aquidauana, and Abobral | Represents the downstream/southern sector, where the flood pulse arrives later because of delayed drainage and hydrological backwater effects along the Paraguay River system. | This sector is affected by delayed flooding, with maximum inundation occurring later than in the north and often overlapping with the beginning of the regional dry season. The area also includes riparian forests, seasonally flooded grasslands, and floodplain habitats where fire and inundation jointly shape vegetation structure. | Fire dynamics in this sector are influenced by delayed hydrological control and distinct seasonal flammability patterns. The overlap between late flooding, subsequent drying, and the dry-season onset can create fire windows that differ from those in the northern and central Pantanal. | Silva and Abdon (1998); Hamilton et al. (1996); Marengo et al. (2021); Stevaux et al. (2020); Arruda et al. (2016) |

**Table S2. Results of the simple regression models between monthly burned area individual predictors. ‘n’ indicates the number of monthly observations used in each regression after accounting for predictor-specific lags.** The table lists the results of the Simple Regression analysis for all predictors tested in relation to monthly BA (log10 ha), ordered in descending order by R². R² quantifies the individual predictive power of each variable. The ‘Model’ column indicates the best-fit function used (Linear or Exponential Saturation), and Slope provides the magnitude and direction of the relationship between the predictor and BA.

| **Variable** | **R²** | **n** | **Slope** | **Model** |
| --- | --- | --- | --- | --- |
| burned_area_lag3 | 0.65 | 480 | 0.95 | Linear |
| precipitation_lag_6m | 0.61 | 480 | -0.01 | Linear |
| BUI_mean_lag_3m | 0.57 | 480 | 0.01 | Exponential Saturation |
| BUI_mean_lag_1m | 0.53 | 480 | 0.02 | Exponential Saturation |
| evapotranspiration_lag_6m | 0.53 | 480 | 0.04 | Linear |
| VPD | 0.53 | 480 | 1.35 | Exponential Saturation |
| VPD_lag_3m | 0.49 | 480 | 1.30 | Exponential Saturation |
| burned_area_lag | 0.45 | 480 | 0.67 | Linear |
| VPD_lag_1m | 0.44 | 480 | 1.19 | Exponential Saturation |
| CAPE_lag_6m | 0.44 | 480 | -0.002 | Linear |
| precipitation_lag_3m | 0.40 | 480 | -0.008 | Linear |
| BUI_mean_lag_6m | 0.38 | 480 | 0.01 | Exponential Saturation |
| precipitation_lag_1m | 0.37 | 480 | -0.007 | Linear |
| CAPE_lag_3m | 0.35 | 480 | -0.001 | Linear |
| wind_gust_lag_3m | 0.35 | 480 | 1.34 | Linear |
| BUI_mean | 0.32 | 480 | 0.01 | Exponential Saturation |
| CAPE_lag_1m | 0.32 | 480 | -0.001 | Linear |
| evapotranspiration_lag_3m | 0.28 | 480 | 0.02 | Linear |
| wind_gust_lag_1m | 0.26 | 480 | 0.92 | Linear |
| evapotranspiration_lag_1m | 0.26 | 480 | 0.01 | Linear |
| wind_gust | 0.25 | 480 | 0.90 | Linear |
| temperature_lag_6m | 0.21 | 480 | -0.35 | Linear |
| burned_area_lag6 | 0.16 | 480 | 0.61 | Linear |
| surface_water | 0.15 | 480 | -0.0000009 | Linear |
| VPD_lag_6m | 0.15 | 480 | 1.06 | Exponential Saturation |
| precipitation | 0.11 | 480 | -0.003 | Linear |
| CAPE | 0.10 | 480 | -0.0006 | Linear |
| SPI_3 | 0.07 | 480 | -0.28 | Linear |
| wind_gust_lag_6m | 0.07 | 480 | 0.84 | Linear |
| precipitation_lag_12m | 0.07 | 480 | -0.01 | Linear |
| precipitation_dev_1yr | 0.07 | 480 | -0.01 | Linear |
| SPI_12 | 0.07 | 480 | -0.28 | Linear |
| SPI_6 | 0.06 | 480 | -0.27 | Linear |
| surface_water_lag_3m | 0.05 | 480 | -0.0000005 | Linear |
| surface_water_lag_1m | 0.04 | 480 | -0.0000005 | Linear |
| temperature_dev_1yr | 0.04 | 480 | 0.35 | Linear |
| temperature_lag_12m | 0.04 | 480 | 0.35 | Linear |
| VPD_dev_1yr | 0.04 | 480 | 0.0006 | Linear |
| VPD_lag_12m | 0.04 | 480 | 0.01 | Linear |
| BUI_mean_dev_1yr | 0.04 | 480 | 0.00002 | Linear |
| BUI_mean_lag_12m | 0.04 | 480 | 0.00009 | Exponential Saturation |
| evapotranspiration | 0.04 | 480 | 0.007 | Linear |
| SPI_1 | 0.04 | 480 | -0.19 | Linear |
| temperature | 0.04 | 480 | 0.08 | Linear |
| precipitation_dev_2yr | 0.03 | 480 | -0.01 | Linear |
| temperature_dev_2yr | 0.03 | 480 | 0.34 | Linear |
| BUI_mean_dev_2yr | 0.02 | 480 | 0.00002 | Linear |

**Table S3. Relative importance of all predictors in the Elastic Net Model for monthly burned area.** The importance of each variable was estimated from the absolute values of the standardized regression coefficients of the Elastic Net model. This value reflects the strength of each predictor's contribution to the variance of the response variable**.**

| **Variable** | **Mean Importance** |
| --- | --- |
| area_lag6 | 0.29 |
| precipitation_lag_1m | 0.28 |
| BUI_mean | 0.20 |
| surface_water | 0.12 |
| area_lag | 0.12 |
| BUI_mean_dev_2yr | 0.11 |
| temperature | 0.09 |
| SPI_3 | 0.08 |
| temperature_dev_1yr | 0.07 |
| wind_gust_lag_1m | 0.07 |
| BUI_mean_lag_1m | 0.07 |
| CAPE | 0.07 |
| surface_water_lag_1m | 0.05 |
| precipitation_dev_1yr | 0.05 |
| CAPE_lag_1m | 0.04 |
| SPI_6 | 0.04 |
| wind_gust | 0.04 |
| SPI_1 | 0.03 |
| BUI_mean_lag_6m | 0.03 |
| wind_gust_lag_6m | 0.03 |
| temperature_dev_2yr | 0.01 |
| Month | 0.00 |
| Year | 0.00 |
| Month:Year | 0.00 |

**Table S4. Cross-Validation and temporal partitioning metrics for the monthly burned area Elastic Net model in the Northern Pantanal.** The table details the training and test partitions used in each of the five folds for the evaluation of the model's temporal stability.

| **Fold** | **Train Period** | **Test Period** | | **Train R²** | | **Test R²** | | **Train RMSE** | **Test RMSE** |
| --- | --- | --- | --- | --- | --- | --- | --- | --- | --- |
| 1 | 1985-2000 | 2000-2004 | 0.82 | | 0.88 | | 0.53 | | 0.37 |
| 2 | 1989-2005 | 2005-2010 | 0.89 | | 0.73 | | 0.38 | | 0.60 |
| 3 | 1994-2011 | 2011-2016 | 0.84 | | 0.90 | | 0.45 | | 0.32 |
| 4 | 1999-2016 | 2017-2020 | 0.88 | | 0.92 | | 0.37 | | 0.29 |
| 5 | 2004-2021 | 2021-2024 | 0.89 | | 0.86 | | 0.36 | | 0.39 |
| Mean | 1985-2021 | 2000-2024 | 0.86 | | 0.86 | | 0.42 | | 0.39 |

**Table S5. Cross-Validation and temporal partitioning metrics for the monthly burned area Elastic Net model in the Central Pantanal.** The table details the training and test partitions used in each of the five folds for the evaluation of the model's temporal stability.

| **Fold** | **Train Period** | **Test Period** | | **Train R²** | | **Test R²** | | **Train RMSE** | **Test RMSE** |
| --- | --- | --- | --- | --- | --- | --- | --- | --- | --- |
| 1 | 1985-2000 | 2000-2004 | 0.70 | | 0.73 | | 0.65 | | 0.45 |
| 2 | 1989-2005 | 2005-2010 | 0.68 | | 0.16 | | 0.62 | | 1.12 |
| 3 | 1994-2011 | 2011-2016 | 0.59 | | 0.82 | | 0.70 | | 0.35 |
| 4 | 1999-2016 | 2017-2020 | 0.57 | | 0.72 | | 0.63 | | 0.53 |
| 5 | 2004-2021 | 2021-2024 | 0.53 | | 0.36 | | 0.69 | | 0.80 |
| Mean | 1985-2021 | 2000-2024 | 0.61 | | 0.56 | | 0.66 | | 0.65 |

**Table S6. Cross-Validation and temporal partitioning metrics for the monthly burned area Elastic Net model in the Southern Pantanal.** The table details the training and test partitions used in each of the five folds for the evaluation of the model's temporal stability.

| **Fold** | **Train Period** | **Test Period** | | **Train R²** | | **Test R²** | | **Train RMSE** | **Test RMSE** |
| --- | --- | --- | --- | --- | --- | --- | --- | --- | --- |
| 1 | 1985-2000 | 2000-2004 | 0.63 | | 0.69 | | 0.71 | | 0.60 |
| 2 | 1989-2005 | 2005-2010 | 0.64 | | 0.52 | | 0.69 | | 0.77 |
| 3 | 1994-2011 | 2011-2016 | 0.66 | | 0.66 | | 0.67 | | 0.58 |
| 4 | 1999-2016 | 2017-2020 | 0.66 | | 0.74 | | 0.64 | | 0.47 |
| 5 | 2004-2021 | 2021-2024 | 0.61 | | 0.48 | | 0.67 | | 0.71 |
| Mean | 1985-2021 | 2000-2024 | 0.64 | | 0.62 | | 0.68 | | 0.63 |

**Table S7. Relative importance of all predictors in the Elastic Net Model for monthly burned area in the Northern Pantanal.** The importance of each variable was estimated from the absolute values of the standardized regression coefficients of the Elastic Net model. This value reflects the strength of each predictor's contribution to the variance of the response variable**.**

| **Variable** | **Mean Importance** |
| --- | --- |
| Burned_area_lag3 | 1.02 |
| burned_area_lag | 0.30 |
| VPD | 0.27 |
| evapotranspiration | 0.15 |
| CAPE | 0.12 |
| BUI_mean_lag_1m | 0.12 |
| precipitation_lag_1m | 0.09 |
| SPI_3 | 0.09 |
| wind_gust_lag_1m | 0.08 |
| VPD_lag_3m | 0.08 |
| wind_gust_lag_6m | 0.07 |
| evapotranspiration_lag_1m | 0.07 |
| BUI_mean_lag_3m | 0.07 |
| precipitation_lag_3m | 0.06 |
| SPI_6 | 0.06 |
| evapotranspiration_lag_3m | 0.06 |
| precipitation | 0.06 |
| VPD_lag_6m | 0.05 |
| surface_water | 0.05 |
| SPI_1 | 0.04 |
| CAPE_lag_6m | 0.04 |
| evapotranspiration_lag_6m | 0.04 |
| SPI_12 | 0.04 |
| CAPE_lag_1m | 0.03 |

**Table S8. Relative importance of all predictors in the Elastic Net Model for monthly burned area in the Central Pantanal.** The importance of each variable was estimated from the absolute values of the standardized regression coefficients of the Elastic Net model. This value reflects the strength of each predictor's contribution to the variance of the response variable**.**

| **Variable** | **Mean Importance** |
| --- | --- |
| burned_area_lag6 | 0.29 |
| VPD | 0.28 |
| surface_water | 0.22 |
| precipitation_lag_1m | 0.17 |
| BUI_mean | 0.10 |
| BUI_mean_dev_2yr | 0.09 |
| CAPE_lag_1m | 0.09 |
| temperature_lag_6m | 0.09 |
| SPI_3 | 0.06 |
| burned_area_lag | 0.06 |
| VPD_lag_1m | 0.05 |
| surface_water_lag_1m | 0.05 |
| BUI_mean_lag_6m | 0.05 |
| BUI_mean_lag_1m | 0.04 |
| wind_gust_lag_1m | 0.04 |
| SPI_12 | 0.03 |
| SPI_6 | 0.02 |
| wind_gust_lag_6m | 0.02 |
| CAPE | 0.02 |
| SPI_1 | 0.02 |
| wind_gust | 0.01 |
| temperature_dev_1yr | 0.01 |
| Year | 0.01 |
| Month | 0.01 |

**Table S9. Relative importance of all predictors in the Elastic Net Model for monthly burned area in the Southern Pantanal.** The importance of each variable was estimated from the absolute values of the standardized regression coefficients of the Elastic Net model. This value reflects the strength of each predictor's contribution to the variance of the response variable**.**

| **Variable** | **Mean Importance** |
| --- | --- |
| burned_area_lag6 | 0.45 |
| VPD | 0.29 |
| surface_water | 0.22 |
| precipitation_lag_1m | 0.12 |
| BUI_mean | 0.12 |
| BUI_mean_dev_2yr | 0.09 |
| CAPE_lag_1m | 0.07 |
| temperature_lag_6m | 0.07 |
| SPI_3 | 0.07 |
| burned_area_lag | 0.06 |
| VPD_lag_1m | 0.05 |
| surface_water_lag_1m | 0.03 |
| BUI_mean_lag_6m | 0.03 |
| BUI_mean_lag_1m | 0.02 |
| wind_gust_lag_1m | 0.02 |
| SPI_12 | 0.01 |
| SPI_6 | 0.01 |
| wind_gust_lag_6m | 0.01 |
| CAPE | 0.00 |
| SPI_1 | 0.00 |
| wind_gust | 0.00 |
| temperature_dev_1yr | 0.00 |
| Year | 0.45 |
| Month | 0.29 |

**References**

Arruda, W. D. S., Oldeland, J., Paranhos Filho, A. C., Pott, A., Cunha, N. L., Ishii, I. H., & Damasceno-Junior, G. A. (2016). Inundation and fire shape the structure of riparian forests in the Pantanal, Brazil. PLoS One, 11(6), e0156825. <https://doi.org/10.1371/journal.pone.0156825>

Hamilton, S. K., Sippel, S. J., & Melack, J. M. (1996). Inundation patterns in the Pantanal wetland of South America determined from passive microwave remote sensing. Archiv für Hydrobiologie, 137, 1–23.

Marengo, J. A., Cunha, A. P., Cuartas, L. A., Deusdará Leal, K. R., Broedel, E., Seluchi, M. E., et al. (2021). Extreme drought in the Brazilian Pantanal in 2019–2020: Characterization, causes, and impacts. Frontiers in Water, 3, 639204. <https://doi.org/10.3389/frwa.2021.639204>

Silva, J. D. S. V., & Abdon, M. M. (1998). Delimitação do Pantanal brasileiro e suas sub-regiões. Pesquisa Agropecuária Brasileira, 33, 1703–1711.

Silva, M. P. D., Mauro, R., Mourao, G., & Coutinho, M. (2000). Distribuição e quantificação de classes de vegetação do Pantanal através de levantamento aéreo. Revista Brasileira de Botânica, 23(2), 143–152. <https://doi.org/10.1590/S0100-84042000000200004>

Stevaux, J. C., de Azevedo Macedo, H., Assine, M. L., & Silva, A. (2020). Changing fluvial styles and backwater flooding along the Upper Paraguay River plains in the Brazilian Pantanal wetland. Geomorphology, 350, 106906. <https://doi.org/10.1016/j.geomorph.2019.106906>

Thielen, D., Schuchmann, K. L., Ramoni-Perazzi, P., Marquez, M., Rojas, W., Quintero, J. I., & Marques, M. I. (2020). Quo vadis Pantanal? Expected precipitation extremes and drought dynamics from changing sea surface temperature. PLoS One, 15(1), e0227437. <https://doi.org/10.1371/journal.pone.0227437>
